# TGF-β signaling in health, disease and therapeutics

**DOI:** 10.1038/s41392-024-01764-w

**Published:** 2024-03-22

**Authors:** Ziqin Deng, Tao Fan, Chu Xiao, He Tian, Yujia Zheng, Chunxiang Li, Jie He

**Affiliations:** Department of Thoracic Surgery, National Cancer Center/National Clinical Research Center for Cancer/Cancer Hospital, Chinese Academy of Medical Sciences and Peking Union Medical College, Beijing, 100021 China

**Keywords:** Immunology, Diseases

## Abstract

Transforming growth factor (TGF)-β is a multifunctional cytokine expressed by almost every tissue and cell type. The signal transduction of TGF-β can stimulate diverse cellular responses and is particularly critical to embryonic development, wound healing, tissue homeostasis, and immune homeostasis in health. The dysfunction of TGF-β can play key roles in many diseases, and numerous targeted therapies have been developed to rectify its pathogenic activity. In the past decades, a large number of studies on TGF-β signaling have been carried out, covering a broad spectrum of topics in health, disease, and therapeutics. Thus, a comprehensive overview of TGF-β signaling is required for a general picture of the studies in this field. In this review, we retrace the research history of TGF-β and introduce the molecular mechanisms regarding its biosynthesis, activation, and signal transduction. We also provide deep insights into the functions of TGF-β signaling in physiological conditions as well as in pathological processes. TGF-β-targeting therapies which have brought fresh hope to the treatment of relevant diseases are highlighted. Through the summary of previous knowledge and recent updates, this review aims to provide a systematic understanding of TGF-β signaling and to attract more attention and interest to this research area.

## Introduction

The studies on TGF-β started as early as the 1980s and have developed rapidly ever since. Although TGF-β was first found to be secreted by transformed cells,^1^ it is widely produced by non-neoplastic tissues such as salivary glands, muscles, kidneys, liver, heart, brain, and embryos as well.^2–4^ In fact, platelets have been identified as one of the most abundant sources of TGF-β among all normal tissues.^5^ The ubiquitous expression of TGF-β in health strongly indicates its critical and multiple roles in physiological conditions.

Accumulating evidence has suggested that TGF-β functions diversely among different cell types in a context-dependent manner. Generally, cell survival, metabolism, growth, proliferation, differentiation, adhesion, migration, and death are all under the regulation of TGF-β. Proper TGF-β signaling is critical to the normal functioning and homeostasis of healthy bodies while aberrant TGF-β signaling can lead to diseases of various categories. For this reason, numerous targeted therapies that can remedy dysregulated TGF-β activity have been developed with some demonstrating encouraging safety and efficacy in clinical trials.

In this review, we focus on the mechanism, physiology, pathology, as well as therapeutics of TGF-β signaling, aiming to provide historical, current, and future perspectives on relevant topics.

## History of research on TGF-β signaling

TGF-β was first reported in 1978 when De Larco and Todaro discovered the ‘sarcoma growth factors’ which were produced by transformed murine fibroblasts and were able to transform normal fibroblasts to anchorage-independent growth.^1^ In 1981, Roberts et al. successfully isolated and purified TGF-β from non-neoplastic murine tissues,^3^ while at about the same time, Moses et al. independently accomplished the purification and characterization of the cytokine as well.^6^ Both groups also noticed that this relatively acid- and heat-stable polypeptide required disulfide bonds for activity and was sensitive to disulfide-reducing agent dithiothreitol. In 1983, studies by electrophoresis on sodium dodecyl sulfate-polyacrylamide gels indicated that the 25,000-dalton TGF-β molecule in humans was actually composed of two 12,500-dalton subunits cross-linked by disulfide bonds.^7,8^ Two years later, the amino-acid sequence of human TGF-β1, the first known TGF-β isoform, was revealed by Derynck et al. through direct protein sequencing and complementary deoxyribonucleic acid (DNA) cloning.^2^ The sequencing established that the 112-amino-acid-long TGF-β1 monomer is initially synthesized as the C-terminal segment of a 390-amino-acid-long precursor polypeptide.^2^ By the time of 1988, researchers had realized that TGF-β generally remained non-covalently associated with the N-terminal segment of its precursor when it was secreted.^9,10^ TGF-β cannot bind to its receptors with its receptor-binding site being masked in this inactive form, however, certain treatments such as acidification could convert latent TGF-β complex into active TGF-β ligand.^11^ In addition, the other two TGF-β isoforms in mammals, TGF-β2 and TGF-β3, were respectively identified in 1987^12^ and 1988.^13,14^ Although the three TGF-β isoforms are encoded by three different genes, their mature ligands show strong conservation of amino acid sequences.

**Fig. 1:**
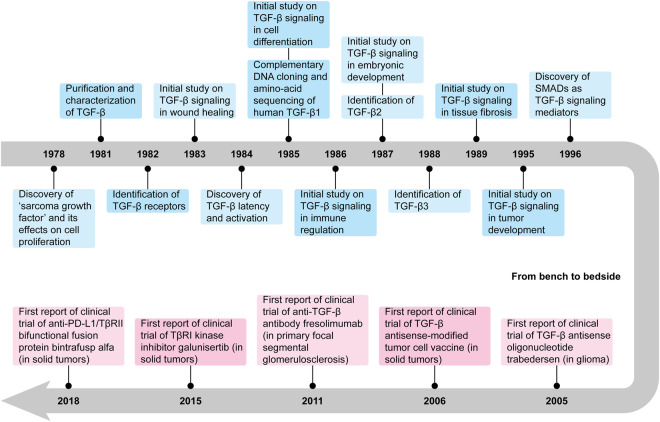
History of research on TGF-β signaling

The effects of TGF-β signaling in cell proliferation,^15,16^ cell differentiation,^17,18^ embryonic development,^19^ wound healing,^20^ immune regulation,^21,22^ tissue fibrosis,^23,24^ and tumor development^25,26^ have been studied shortly after the discovery of the cytokine. Meanwhile, the receptors in TGF-β signaling known as TGF-β receptor I (TβRI) and TβRII were also identified and characterized in the 1980s.^27–29^ But it was not until the discovery of signaling mediators small (Sma) in *Caenorhabditis elegans* and mothers against decapentaplegic (Mad) in *Drosophila melanogaster* that the homologous small mothers against decapentaplegic (SMAD) proteins were identified as the canonical signal transducers of TGF-β signaling in humans in 1996.^30–32^ Since then, the development of TGF-β research has been largely accelerated. In recent times, as studies on TGF-β signaling in both health and disease going deeper and further, a lot of TGF-β-targeting therapies have been developed and assessed for the treatment of various diseases,^33–39^ revealing a promising future for the studies in this area **(**Fig. 1**)**.

## Biosynthesis and activation of TGF-β

**Fig. 2:**
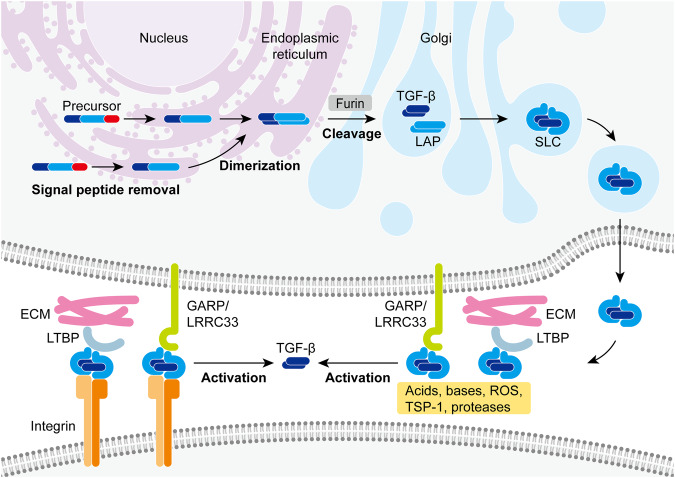
Biosynthesis and activation of TGF-β. Each TGF-β monomer is initially synthesized as a precursor polypeptide. In the endoplasmic reticulum, TGF-β precursors lose their signal peptides and dimerize through disulfide bonds. The dimers then transit into the Golgi where they are cleaved by protease furin into mature cytokine segments and latency-associated peptides (LAPs) to form small latent complexes (SLCs). The secreted SLCs can further link to latent TGF-β-binding proteins (LTBPs) which target them into the extracellular matrix (ECM) for storage, or they can link to glycoprotein-A repetitions predominant protein (GARP) or leucine-rich repeat-containing protein 33 (LRRC33) which tethers them to the cell surface. Numerous factors such as acids, bases, reactive oxygen species (ROS), thrombospondin-1 (TSP-1), certain proteases, and integrins can release the mature cytokines from the latent complexes and thus are known as TGF-β activators

During the biosynthesis of TGF-β, the precursor undergoes post-translational processing to become a latent complex which is the secretory form of TGF-β. The latent TGF-β complex still requires further activation to eventually become a mature cytokine before it can trigger signal transduction in cells (Fig. 2).

### TGF-β biosynthesis and latency

Each TGF-β monomer is initially synthesized as a precursor polypeptide composed of a mature cytokine as its C-terminal segment, a signal peptide at the N-terminus, and a latency-associated peptide (LAP) in between.^2^ The signal peptide leads the precursor into the endoplasmic reticulum lumen and promptly gets removed. The remainder of the precursor then dimerizes through three disulfide bonds and transits into the Golgi where it gets cleaved between the mature cytokine and LAP by protease furin.^40^ However, the cytokine segment is still unable to bind its receptors after the cleavage, for it remains associated with LAP in a non-covalent way that masks its receptor-binding site and forms a small latent complex (SLC).^41^ In most cases, LAP is linked to latent TGF-β-binding protein (LTBP) through a disulfide bond, making the SLC into a large latent complex (LLC) when secreted.^42^ LTBP can further bind to fibrillin to target the LLC into the extracellular matrix (ECM) for storage.^43^ Alternatively, LAP can also form disulfide linkage with leucine-rich repeat-containing protein 32 (LRRC32) or LRRC33 to tether SLC to the cell surface. Unlike LTBP which is widely expressed by many cell types, LRRC32, also known as glycoprotein-A repetitions predominant protein (GARP), is specifically detected in regulatory T cells (Tregs), platelets, and endothelium,^44^ whereas high expression of LRRC33 is found in macrophages, dendritic cells (DCs), and B cells.^45^

### TGF-β activation

The bioactivity of TGF-β is based on ligand-receptor interaction which requires the exposure of its receptor-binding site. Thus, the activation of TGF-β represents the release of mature cytokine from the latent complex. Numerous factors have been identified as TGF-β activators as introduced below. Notably, integrin-dependent activation is so far the best described and likely the most important mechanism, while TGF-β activation mediated by acids, bases, reactive oxygen species (ROS), thrombospondin-1 (TSP-1), proteases, and other TGF-β activators is collectively known as integrin-independent activation.

#### TGF-β activation by integrins

Integrins are heterodimeric transmembrane receptors each consisting of an α-subunit and a β-subunit. TGF-β activation by integrins requires the binding of the integrins to an RGD sequence in the LAP of TGF-β1 and TGF-β3. Therefore, latent TGF-β2 without the RGD motif is excluded from integrin-dependent activation.^46^

Among all integrins, αVβ6 and αVβ8 integrins are the best studied TGF-β activators. The expression of αVβ6 integrin is nearly restricted to epithelial cells and is upregulated in response to morphogenesis, wounding, inflammation, and tumorigenesis.^47^ In contrast, αVβ8 integrin is widely expressed by epithelial cells,^48^ fibroblasts,^49^ macrophages,^50^ DCs,^51^ Tregs,^52^ and different kinds of tumor cells.^53^ The lack of αVβ6 and αVβ8 integrin activity reproduces the phenotypes of TGF-β1- and TGF-β3-null mice, indicating the central importance of integrin-dependent activation.^54,55^

Upon binding to the RGD motif in LAP, the mechanisms by which αVβ6 and αVβ8 integrins activate TGF-β are quite different. With latent TGF-β being tethered to ECM or cell membrane (through the binding of LAP to LTBP, GARP, or LRRC33 as mentioned before) and the cytoplasmic domain of integrin β6 subunit linking to the actin cytoskeleton, αVβ6 integrin can transmit contractile force which changes the conformation of LAP to release TGF-β ligand.^56,57^ However, the cytoplasmic domain of integrin β8 subunit does not link to the actin cytoskeleton. One effective mechanism for αVβ8 integrin-mediated TGF-β activation requires the proteolytic activity of membrane type 1-matrix metalloproteinase (MT1-MMP, also known as MMP14).^48^ Alternatively, membrane molecules such as GARP and LRRC33 which bind and present latent TGF-β on the surface of one cell can cooperate with the αVβ8 integrin expressed on a different cell to activate TGF-β in trans.^45,58,59^ A recent study reveals that upon binding to αVβ8 integrin, the flexible membrane-presented latent complex can expose the active domain of the TGF-β ligand to its receptors for binding and signaling without the need to release diffusible cytokine.^60^

#### TGF-β activation by acids and bases

It has long been noticed that acidification can unmask the activity of freshly secreted TGF-β.^61^ Sharply defined parameters for human TGF-β activation by acids and bases show that the transition from latency of all three isoforms occurred between pH 2.5 and 4, and between pH 10 and 12.^62^ Thus, extremely acidic environments such as the microenvironments in tumor tissues and the resorption lacunae of osteoclasts are possibly conducive to local TGF-β activation.^63,64^ A study on lung fibrosis even suggests that physiologic concentrations of lactic acid are sufficient enough to activate TGF-β in a pH-dependent manner.^65^

#### TGF-β activation by ROS

TGF-β1 is the only isoform that can be directly activated by ROS, for a unique methionine residue at the amino acid position 253 of its LAP is required for oxidation-triggered conformational change.^66^ However, ROS can induce other TGF-β activators such as TSP-1^67^ and MMPs^68^ to activate all three isoforms in an indirect manner. ROS-mediated TGF-β activation prevails in tissues exposed to asbestos,^69,70^ ultraviolet,^68^ and ionizing radiation.^71^ High glucose intake can also induce ROS production and consequentially increase TGF-β activation to play roles in the development of fibrotic diseases and inflammatory diseases.^72,73^ Moreover, in T cells, ROS can be elevated during apoptosis or upon stimulation by T cell receptor (TCR) and cluster of differentiation 28 (CD28) to contribute to the immunosuppression mediated by activated TGF-β.^74,75^

#### TGF-β activation by TSP-1

TSP-1 is a multi-functional ECM protein not only abundant in platelet α-granules but also secreted by fibroblasts, endothelial cells, macrophages, T cells, and many other cell types.^76^ The KRFK sequence in TSP-1 can recognize the LSKL sequence in LAP to competitively disrupt its interaction with the receptor-binding site of the TGF-β ligand. Since the LSKL sequence in LAP is conserved among TGF-β isoforms, it is suggested that the direct binding of TSP-1 to latent complex is capable of activating all three TGF-β isoforms through this protease- and cell-independent mechanism.^77^ Interestingly, TSP-1 can also bind to the mature TGF-β ligand to form a complex that retains the biological activity of the cytokine.^78^ ROS,^67^ glucose,^79^ angiotensin II,^80^ hypoxia,^81^ wounding,^82^ inflammation,^83^ pathogens,^84–86^ and many other factors can all induce TSP-1 to function as a TGF-β activator in wound healing,^67,82^ cardiovascular diseases,^81,86^ renal diseases,^79^ fibrotic diseases,^87,88^ inflammatory diseases,^83^ infectious diseases,^89^ and tumors.^90^

#### TGF-β activation by proteases

Many proteases have been proved capable of directly activating TGF-β in vitro. However, the function of an individual protease seems redundant in vivo, as deficiency of a single species generally leads to no significant signs of impaired TGF-β activation.^91^ Among these proteases, MMPs such as MMP-2, MMP-9, and MMP-13 are conducive to the TGF-β activation in wound healing,^92^ cardiovascular diseases,^93^ renal diseases,^94^ fibrotic diseases,^95^ and tumors.^96^ Interestingly, although the activation by MMPs works for all three TGF-β isoforms, latent TGF-β2 and TGF-β3 appear much more sensitive to MMP-9 treatment than latent TGF-β1.^96^ Moreover, a serine protease known as plasmin plays an important role in the TGF-β activation mediated by macrophages^97,98^ and endothelial cells.^99,100^

## Signal transduction of TGF-β

TGF-β signal is transmitted into the cells by TβRI (also known as activin receptor-like kinase 5, ALK5) and TβRII both of which are enzyme-linked receptors with dual specificity of serine/threonine kinase and tyrosine kinase. Studies have revealed that TGF-β1 and TGF-β3 bind TβRII prior to TβRI due to higher affinity, while TGF-β2 binds poorly to both receptors.^12,101,102^ TβRIII, also known as β-glycan, lacks the motifs to directly mediate TGF-β signal transduction. However, TβRIII is able to bind TGF-β especially TGF-β2 with high affinity and thus acts as a co-receptor that presents the ligand to the receptors and further enhances their binding.^101,103–107^ The ligand-receptor interaction subsequently activates the intracellular signaling of TGF-β through a canonical pathway and several non-canonical pathways.

### Canonical TGF-β signaling

**Fig. 3:**
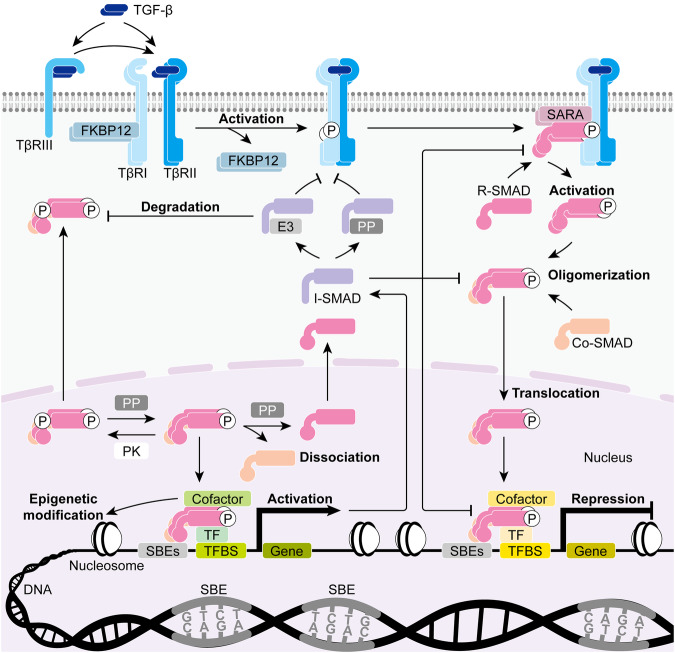
Canonical TGF-β signaling. TGF-β can initially bind to its co-receptor TGF-β receptor III (TβRIII) or directly bind to its receptor TβRII which subsequently recruits TβRI to form a TGF-β-TβRI-TβRII complex. TβRII then actives TβRI through phosphorylation, leading to its dissociation with signaling inhibitor FK506-binding protein 1A (FKBP12) and interaction with signaling effectors receptor-activated SMADs (R-SMADs). R-SMADs which are presented to TβRI by adaptor protein SMAD anchor for receptor activation (SARA) get activated through phosphorylation and undergo oligomerization with common-partner SMAD (co-SMAD). The SMAD oligomers then translocate into the nucleus where they function as transcription factors (TFs), mediating the transcriptional activation or repression of target genes by binding to specific DNA sequences known as SMAD-binding elements (SBEs) and generally in cooperation with other TFs as well as transcriptional cofactors. In this way, TGF-β signaling can activate the expression of inhibitory SMADs (I-SMADs) which in turn function to attenuate the transcriptional regulation mediated by TGF-β signaling through several mechanisms. Moreover, many protein kinases (PKs), protein phosphatases (PPs), and (E3) ubiquitin ligases can also modulate canonical TGF-β signaling through various post-translational modifications of SMADs. (TFBS, TF-binding site)

The canonical TGF-β signaling is mediated by transcription factors SMADs and thus is also known as the SMAD signaling. Notably, the canonical pathway is under the regulation of various factors that can control the intensity and manner of cellular responses at different levels (Fig. 3).

#### TGF-β-activated SMAD signaling

TGF-β ligand initially binds to TβRII monomer to promote its homodimerization or directly binds to pre-existing TβRII homodimer to recruit TβRI for assembly.^108–111^ This forms a heteromeric TGF-β-TβRI-TβRII complex in which low-affinity TβRI requires high-affinity TβRII to bind TGF-β ligand and constitutively active TβRII requires phosphorylating TβRI to transduce intracellular signal.^112^ The phosphorylation of TβRI occurs in its juxtamembrane GS domain at several serine and threonine residues, triggering conformational changes that transform the GS domain from a site that binds the signaling inhibitor known as immunophilin FK506-binding protein 1A (FKBP12) into a binding site for the signaling effectors known as receptor-activated SMADs (R-SMADs).^113^

R-SMADs, including SMAD2 and SMAD3, consist of a globular Mad homology 1 (MH1) domain at the N-terminus, a globular MH2 domain at the C-terminus, and a highly flexible long linker region in between. R-SMADs are retained in cytoplasm and presented to TβRI by the adaptor protein known as SMAD anchor for receptor activation (SARA).^114^ The R-SMAD MH2 domain then gets phosphorylated at two serine residues in the extreme C-terminal SXS motif by the TβRI kinase domain which is located immediately downstream of the TβRI GS domain.^113^ Activated R-SMADs undergo homo-oligomerization or hetero-oligomerization through their MH2 domains upon phosphorylation, and they can also oligomerize with SMAD4, the common-partner SMAD (co-SMAD) which lacks the SXS motif for phosphorylation by TβRI kinase. Notably, studies have suggested that SMAD heterotrimers containing two R-SMADs and one SMAD4 are likely more common and stable than other SMAD oligomers.^115–119^ Although different SMAD oligomers can vary in function, they all act to regulate the transcription of target genes by binding to DNA after translocating into the nucleus. The MH1 domains of SMAD4, SMAD3, and a specific SMAD2 splicing variant recognize the nucleic acid sequence GTCT or its reverse complement AGAC in double-stranded DNA which are known as the canonical SMAD-binding elements (SBEs).^120^ Other SBEs such as the 5GC SBEs including GGCGC and GGCCG have also been discovered, indicating a relatively loose DNA-binding specificity of the SMAD oligomers.^121^ However, the binding to a single SBE is so weak that SMAD oligomers generally require interacting with replications of SBE copies as well as other DNA-binding sequence-specific transcription factors to function.^119,120,122^ In fact, many SBE repeats are enriched at the binding sites for SMAD-interacting transcription factors, exactly increasing the binding accessibility, specificity, and affinity of SMAD oligomers associated with specific transcription factors.^123–125^ Despite a large number of SMAD-interacting transcription factors indicating a huge amount of potential gene targets for canonical TGF-β signaling, the dominant effects are generally determined by the master transcription factors in specific cell types and contexts which contribute to the complexity and variability of cellular responses to TGF-β.^125^

#### Regulation of SMAD signaling by inhibitory SMADs (I-SMADs)

TGF-β and many other factors can induce the expression of SMAD6 and SMAD7 which function to inhibit TGF-β signaling and thus are known as I-SMADs.^126,127^ Unlike R-SMADs, I-SMADs lack the N-terminal MH1 domain and the C-terminal SXS motif, however, they retain the C-terminal MH2 domain which can competitively bind to activated receptor TβRI to inhibit the phosphorylation of R-SMADs.^128,129^ Through some extra mechanisms, SMAD7 confers greater abilities in suppressing TGF-β signaling than SMAD6 does.^130^ For example, SMAD7 recruits E3 ubiquitin ligases such as SMAD ubiquitination regulatory factors (SMURFs) and neural precursor cell expressed, developmentally downregulated 4-like (NEDD4L) to TβRI, R-SMADs, and co-SMAD to mediate the proteasomal and lysosomal degradation of these TGF-β signaling components.^131–135^ SMAD7 can also trigger the dephosphorylation of TβRI by recruiting protein phosphatase 1 (PP1) to the receptor.^136^ Moreover, with its MH2 domain, SMAD7 can oligomerize with R-SMADs to compete with co-SMAD^133^ and can bind to specific DNA sequences to disrupt the formation of the transcriptional SMAD-DNA complex.^137^ Taken together, TGF-β signaling induces I-SMADs to form a negative feedback loop of itself.

#### Regulation of SMAD signaling by transcriptional cofactors

Transcriptional cofactors are actively recruited to the transcriptional SMAD complex to regulate its activity. Notably, many of these transcriptional cofactors have histone modification activity and thus enable TGF-β signaling to trigger epigenetic changes. Histone acetyltransferases (HATs) such as p300, cyclic adenosine monophosphate (cAMP) response element-binding protein (CREB)-binding protein (CBP), p300/CBP-associated factor (PCAF), and general control non‐repressed protein 5 (GCN5) act as the transcriptional coactivators of SMADs by increasing the accessibility to DNA.^138–141^ The interaction between p300/CBP and doubly phosphorylated R-SMADs requires SMAD4 for stabilization and is critical for SMAD-mediated transcriptional activation. Other SMAD coactivators include melanocyte-specific gene 1 (MSG1),^142^ zinc finger E-box-binding homeobox 1 (ZEB1),^143,144^ and the histone methyltransferase (HMT) known as SET domain-containing protein 7 (SETD7).^145^ Contrary to HATs, histone deacetylases (HDACs) generally act as the transcriptional corepressors of SMADs by decreasing the accessibility to DNA. SMAD3 can directly recruit HDAC4 and HDAC5 to gene promoters to inhibit the function of transcription factors via histone deacetylation.^146^ SMADs can also associate with HDACs through interaction with other corepressors such as TGF-β-induced factor (TGIF),^147^ ecotropic viral integration site 1 (EVI1),^148,149^ Sloan-Kettering Institute proto-oncogene (SKI),^150–152^ as well as SKI-related novel gene N (SNO).^153^ Other transcriptional corepressors of SMADs include cellular-myelocytomatosis viral oncogene (MYC),^154^ SMAD nuclear-interacting protein 1 (SNIP1),^155^ ZEB2,^143,156^ and HMTs such as suppressor of variegation 3-9 homolog 1 (SUV39H1) and SET domain bifurcated 1 (SETDB1) which can both trigger the methylation of histone 3 lysine 9 (H3K9) at gene promoters.^157,158^

#### Regulation of SMAD signaling by SMAD modifications

Post-translational modifications can also regulate the functions of SMADs. Apart from TβRI kinase which phosphorylates R-SMADs in their C-terminal SXS motif to mediate their activation, many other protein kinases such as mitogen-activated protein kinase kinase kinase 1 (MAPKKK1),^159^ p38 MAPK,^160^ c-Jun N-terminal kinase (JNK),^161^ extracellular signal-regulated kinase (ERK),^162–164^ rat sarcoma (RAS) homolog (Rho)-associated coiled-coil-containing protein kinase (ROCK),^160^ glycogen synthase kinase (GSK)-3β,^165–167^ calcium/calmodulin-dependent protein kinase II (CAMK2),^168^ protein kinase C (PKC),^169^ PKG,^170^ and several cyclin-dependent kinases (CDKs)^167,171,172^ can phosphorylate R-SMADs as well as co-SMAD at many different sites to enhance or attenuate SMAD activity. Meanwhile, the various phosphorylation of SMADs can be reversed by phosphatases. Several nuclear phosphatases known as the small C-terminal domain phosphatases (SCPs) can specifically dephosphorylate the linker region and MH1 domain of R-SMADs,^173,174^ whereas protein phosphatase, magnesium/manganese-dependent 1A (PPM1A),^175^ myotubularin-related protein 4 (MTMR4),^176^ and protein phosphatase 2A (PP2A)^177^ catalyze the dephosphorylation of the C-terminal SXS motif to terminate the signaling and promote the dissociation and cytoplasmic localization of SMADs.

Furthermore, SMADs can be ubiquitinated and deubiquitinated respectively by E3 ubiquitin ligases and deubiquitylating enzymes (DUBs). The E3 ubiquitin ligases that can mediate SMAD ubiquitination include SMURFs,^135,178–180^ NEDD4L,^134,181^ WW domain-containing proteins (WWPs),^182–184^ really interesting new gene (RING) finger protein 111 (RNF111),^185^ C-terminus of heat shock protein (HSP) 70-interacting protein (CHIP),^186^ itchy (ITCH) E3 ubiquitin ligase,^187^ and S-phase kinase-associated protein (SKP)-cullin-F-box (SCF) E3 ubiquitin ligase complex.^188,189^ The ubiquitination generally leads to the proteasomal degradation of SMADs, but in some cases, it also exerts non-degradative effects on SMAD activity.^190^ Notably, the degradative ubiquitination of R-SMADs by NEDD4L requires the phosphorylation of the R-SMAD linker by CDK8/9 and GSK-3 in sequence to create binding sites for the E3 ubiquitin ligase.^171,181,191^

### Non-canonical TGF-β signaling

**Fig. 4:**
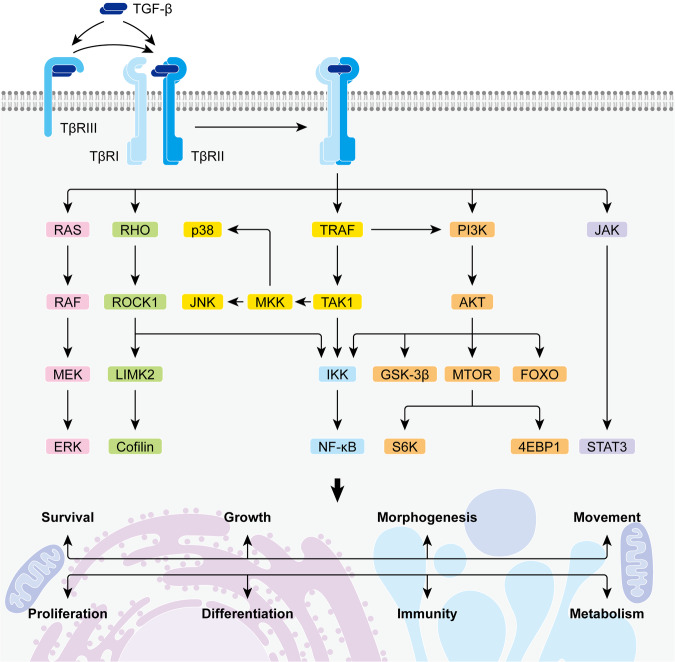
Non-canonical TGF-β signaling. TGF-β can signal through non-canonical pathways to activate extracellular signal-regulated kinase (ERK) signaling, rat sarcoma (RAS) homolog (Rho)-guanosine triphosphatase (GTPase) signaling, p38 mitogen-activated protein kinase (MAPK) signaling, c-Jun N-terminal kinase (JNK) signaling, nuclear factor-κB (NF-κB) signaling, phosphatidylinositol 3-kinase (PI3K)/AKR mouse thymoma proto-oncogene (AKT) signaling, as well as Janus kinase (JAK)/signal transducer and activator of transcription (STAT) signaling. These non-canonical TGF-β signaling pathways are actively involved in an extensive range of cellular events. (RAF, RAS-associated factor; MEK, MAPK/ERK kinase; ROCK1, Rho-associated coiled-coil-containing protein kinase 1; LIMK2, LIM domain kinase 2; TRAF, tumor necrosis factor (TNF) receptor-associated factor; TAK1, TGF-β-activated kinase 1; MKK, MAPK kinase; IKK, NF-κB inhibitor (IκB) kinase; GSK-3β, glycogen synthase kinase-3β; MTOR, mechanistic target of rapamycin; FOXO, forkhead box O; S6K, S6 kinase; 4EBP1, 4E-binding protein 1)

Apart from the SMAD-dependent pathway, TGF-β can also signal through SMAD-independent pathways to activate ERK signaling, Rho guanosine triphosphatase (GTPase) signaling, p38 MAPK signaling, JNK signaling, nuclear factor-κB (NF-κB) signaling, phosphatidylinositol 3-kinase (PI3K)/AKR mouse thymoma proto-oncogene (AKT) signaling, as well as Janus kinase (JAK)/signal transducer and activator of transcription (STAT) signaling. These non-canonical TGF-β signaling pathways are involved in an extensive range of cellular events, greatly expanding the participation of TGF-β signaling in health and disease (Fig. 4).

#### TGF-β-activated ERK signaling

As a dual-specificity kinase, TβRI can phosphorylate at its tyrosine residues to activate ERK signaling upon TGF-β stimulation.^192^ In this case, TβRI with tyrosine kinase activity initially phosphorylates the adapter protein known as sarcoma (SRC) homology and collagen A (SHCA) which subsequently forms a complex with growth factor receptor-bound protein 2 (GRB2) and son of sevenless homolog (SOS). The SHCA-GRB2-SOS complex then initiates a canonical MAPK signaling cascade which involves the sequential activation of RAS, the MAPKKK known as RAS-associated factor (RAF), the MAPKK known as MAPK/ERK kinase (MEK), and eventually, the ERK MAPK. Activated ERK is known to regulate various biological events including cell survival, proliferation, differentiation, adhesion, migration, as well as metabolism, and is implicated in a spectrum of diseases such as developmental disorders, chronic inflammation, neurodegeneration, obesity, and cancers.^193,194^

#### TGF-β-activated Rho GTPase signaling

Rho GTPases such as RHO, RAS-related C3 botulinum toxin substrate 1 (RAC1), and cell division cycle 42 (CDC42) play a central role in the organization and dynamics of the actin cytoskeleton. They are activated by guanine nucleotide exchange factors (GEFs) through the exchange of a bound GDP for GTP.^195^ TGF-β can trigger RHO activation in a rapid SMAD-independent manner or by inducing a GEF known as neuroepithelial cell transforming 1 (NET1) through SMAD and MEK/ERK pathways.^196–200^ RHO then activates its key effector ROCK1 which further mediates the phosphorylation of LIM domain kinase 2 (LIMK2). Activated LIMK2 subsequently phosphorylates cofilin to inhibit its function as a constitutive actin-depolymerizing factor, leading to the reorganization of the actin cytoskeleton in the end.^201–203^ Additionally, TGF-β-triggered RHO/ROCK1 signaling can contribute to ERK phosphorylation,^204,205^ and besides RHO, TGF-β can also activate the signaling of other Rho GTPases such as RAC1^202^ and CDC42.^206^ Besides the regulation of cell morphogenesis, adhesion, and movement, Rho GTPase signaling is also known to participate in transcriptional regulation, cell cycle progression, vesicular trafficking, and pathological processes such as fibrosis, inflammation, wound repair, and tumor development.^207,208^

#### TGF-β-activated p38, JNK, and NF-κB signaling

TGF-β can activate the signaling of another two MAPKs known as p38 and JNK through a receptor kinase-independent mechanism which is different from that of ERK signaling. TGF-β-activated TβR complex can recruit tumor necrosis factor (TNF) receptor-associated factor 4 (TRAF4) and TRAF6 to trigger their lysine 63 (K63)-linked polyubiquitination. With E3 ubiquitin ligase activity, polyubiquitinated TRAF then attaches the polyubiquitin chain on the MAPKKK known as TGF-β-activated kinase 1 (TAK1) which subsequently gets activated and phosphorylates several MAPKKs (MKKs).^209–211^ As a result, MKK3 and MKK6 specifically trigger the activation of p38 while MKK4 mediates the phosphorylation of both p38 and JNK. TGF-β-activated Rho GTPases such as RHOA, RAC1, and CDC42 can also contribute to p38 and JNK activation.^204,212–216^ Both the two MAPKs regulate a series of biological events to respond to all kinds of environmental and intracellular stresses, meanwhile, they engage actively in embryonic development, metabolic regulation, neuronal functions, immunological actions, as well as tumor development.^217–220^

Additionally, TGF-β-activated TRAF/TAK1 signaling, RHO/ROCK1 signaling, and PI3K/AKT signaling can also lead to the phosphorylation of NF-κB inhibitor (IκB) kinase (IKK).^221–224^ Activated IKK then triggers the phosphorylation of IκB which subsequently gets polyubiquitinated and degraded while releasing active NF-κB for nuclear translocation.^221^ NF-κB as a transcription factor can regulate hundreds of genes involved in cell survival, proliferation, metabolism, and immunity in particular.^225–227^

#### TGF-β-activated PI3K/AKT signaling

The TβR complex can activate the lipid kinase PI3K upon TGF-β stimulation, either via the kinase activity of TβRI or through the recruitment of TRAF6, which polyubiquitylates PI3K regulatory subunit p85α independent of the receptor kinase.^228,229^ Activated PI3K then phosphorylates phosphoinositide phosphatidylinositol-4,5-bisphosphate (PIP2) into phosphatidylinositol-3,4,5-trisphosphate (PIP3) which further triggers the phosphorylation of AKT.^228,230^ Activated AKT targets plenty of substrates, including mechanistic target of rapamycin (MTOR),^231,232^ GSK-3β,^233^ and several forkhead box O (FOXO) transcription factors.^234^Among them, MTOR is the most common downstream effector of AKT, and ribosomal protein S6 kinase (S6K) and eukaryotic initiation factor 4E-binding protein 1 (4EBP1) are the best-characterized downstream effectors of MTOR. In general, the consequences of PI3K/AKT signaling include diverse cellular responses such as survival, metabolism, growth, proliferation, and differentiation.^235^

#### TGF-β-activated JAK/STAT signaling

TGF-β is found to induce JAK1 and JAK2 activation respectively in hepatic stellate cells (HSCs) and fibroblasts. In these cases, activated JAK triggers the phosphorylation of STAT3 which functions to mediate the fibrogenic effects of TGF-β, including increased cell proliferation, myofibroblast (MF) differentiation, ECM production, α-smooth muscle actin (α-SMA) expression, and stress fiber formation.^236–238^ Like other signaling pathways, JAK/STAT signaling can also drive many physiological and pathological events, including development, metabolism, immunity, wounding, and cancers.^239^

## TGF-β signaling in health

**Fig. 5:**
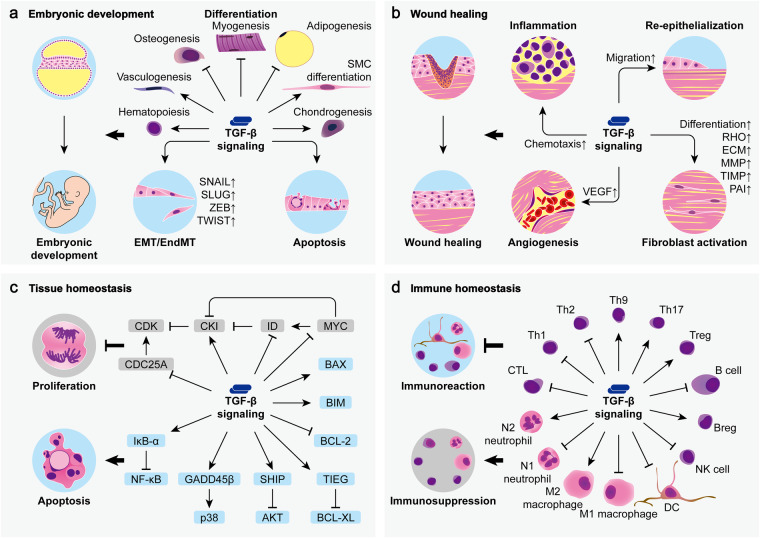
TGF-β signaling in health. TGF-β signaling plays a critical role in physiological conditions. **a** During embryonic development, TGF-β regulates cell differentiation, epithelial/endothelial-mesenchymal transition (EMT/EndMT), and apoptosis to ensure proper histogenesis and organogenesis. **b** TGF-β promotes wound healing by participating in inflammation, re-epithelialization, angiogenesis, and fibroblast activation. **c** TGF-β is indispensable for tissue homeostasis as it generally suppresses cell proliferation and induces cell apoptosis through various mechanisms. **d** TGF-β functions to suppress the activity of multiple immunocompetent cells while inducing the phenotypes of several immune immunosuppressive cells to maintain immune homeostasis. (SMC, smooth muscle cell; VEGF, vascular endothelial growth factor; MMP, matrix metalloproteinase; TIMP tissue inhibitor of MMP, PAI plasminogen activator inhibitor, CDK cyclin-dependent kinase, CKI CDK inhibitor, ID inhibitor of DNA binding, MYC cellular-myelocytomatosis viral oncogene, CDC25A cell division cycle 25A, BCL-2 B-cell lymphoma-2, BAX BCL-2-associated X protein, BIM BCL-2-interacting mediator of cell death, BCL-XL BCL-extra-large, GADD45β growth arrest and DNA damage-inducible β, SHIP sarcoma (SRC) homology 2 (SH2) domain-containing inositol 5’-phosphatase, TIEG TGF-β-inducible early gene, CTL cytotoxic T lymphocyte, Th T helper, Treg regulatory T cell, Breg regulatory B cell, NK natural killer, DC dendritic cell)

In physiological conditions, TGF-β signaling is greatly required by multiple biological processes and is particularly critical to embryonic development, wound healing, tissue homeostasis, and immune homeostasis (Fig. 5).

### Embryonic development

In situ hybridization and immunohistochemical staining reveal overlapping but distinct expression patterns of the three TGF-β isoforms at different developmental stages of murine embryos. TGF-β is expressed in nearly all kinds of embryonic tissues such as heart, vessels, lungs, kidneys, liver, gut, bones, teeth, cartilages, muscles, skin, thymus, thyroid, suprarenal glands, salivary glands, nervous system, and craniofacial tissues.^19,240–244^ In particular, mesenchymal and epithelial components undergoing organogenesis and morphogenesis which involve active cell differentiation and epithelial-mesenchymal interactions generally express high levels of TGF-β.^19,240–243^

TGF-β has a significant impact on cell differentiation. Studies on *Xenopus* embryos reveal that TGF-β can induce mesoderm formation which is a primary patterning event in early vertebrate development.^245,246^ TGF-β can further regulate the development of hemangioblasts from mesoderm as well as subsequent differentiation of hematopoietic stem and progenitor cells (HSPCs) to participate in hematopoiesis and vasculogenesis.^240,247–250^ Mesenchymal stem cells (MSCs) which are derived from the mesoderm as well also respond actively to TGF-β signaling during their differentiation into several connective tissue cell lineages such as osteocytes, chondrocytes, myocytes, and adipocytes.^251,252^ TGF-β inhibits osteogenic differentiation by inducing the nuclear translocation of β-catenin and repressing the transcriptional activity of core-binding factor subunit α-1 (CBFA1) in a SMAD3-dependent manner.^252,253^ TGF-β-induced SMAD signaling also inhibits myogenesis and adipogenesis by respectively repressing the transcriptional activity of myogenic differentiation (MYOD) family members^254–257^ and CCAAT/enhancer-binding proteins (C/EBPs).^17,258,259^ However, the differentiation of MSCs into smooth muscle cells (SMCs) is promoted by TGF-β through mechanisms involving the activation of SMAD signaling, RHO signaling, and NOTCH signaling.^260^ Moreover, TGF-β stimulates chondrogenesis by inducing mesenchymal cells to differentiate into chondrocytes and produce cartilage-specific proteoglycan and type II collagen.^18,261,262^ As for other cell types, TGF-β signaling also regulates the differentiation and development in epidermis,^263^ lungs,^264,265^ kidneys,^266^ pancreas,^267,268^ teeth,^269^ and nervous system.^270–276^

Especially for epithelial cells, TGF-β can induce a reversible de-differentiation process known as epithelial-mesenchymal transition (EMT) which is critical to embryonic development.^277^ During EMT, epithelial cells lose their cellular polarity, intercellular junctions, and epithelial markers such as E-cadherin, but turn to acquire mesenchymal or fibroblastic phenotype with increased cell migratory motility, ECM proteolytic activity, and expression of mesenchymal markers such as fibronectin.^278^ This process is generally mediated by transcription factors such as SNAIL, SLUG, ZEB, and TWIST, involving both SMAD-dependent and SMAD-independent pathways in the case of TGF-β signaling.^198,200,219,230–232,279,280^ The developmental functions of TGF-β-induced EMT have been well studied in embryonic palate formation during which the expression of TGF-β is significantly elevated.^19,243^ Among the three TGF-β isoforms expressed in developing murine palate,^281,282^ only TGF-β3 is indispensable to the fusion of palatal shelves which is a crucial step during palatogenesis.^283^ Mechanically, TGF-β3 induces the EMT of palatal midline epithelial seam (MES) cells, leading to the disintegration of the epithelium and subsequent confluence of the mesenchyme.^279,280^ Interestingly, endothelial cells can undergo a similar process known as endothelial-mesenchymal transition (EndMT) which is crucial for cardiovascular development. In humans, TGF-β2 is the most potent inducer of EndMT, while TGF-β1 and TGF-β3 at least partially rely on the induction of TGF-β2 to trigger this process.^284^ Consistently, although all three TGF-β isoforms are differentially expressed during murine cardiogenesis,^19,240,242,243,285–287^ only TGF-β2 is obligatory to the EndMT during the endocardial cushion development in the atrioventricular canal which is necessary to valvular formation.^288–291^ Moreover, TGF-β1 and TGF-β2 can trigger EndMT in the epicardium to contribute to coronary vessel formation.^292,293^ In fact, TGF-β signaling is essential to vasculogenesis in many developing tissues by promoting the proliferation and migration of endothelial cells.^19,294^

Furthermore, TGF-β can induce apoptosis of unnecessary cells during embryonic development to ensure proper histogenesis and organogenesis. During murine palatogenesis, the disintegration of MES not only relies on TGF-β3-induced EMT as introduced above but also requires TGF-β3-induced apoptosis of MES cells to complete palatal confluency.^295^ In murine limb buds, highly expressed TGF-β triggers massive cell death in the mesenchyme of interdigital spaces to induce the regression of interdigital webs and the formation of free digits.^19,243,296^ Endogenous TGF-β also mediates the apoptotic death of certain neuron types in chick embryos to contribute to nervous system development.^297^ Notably, TGF-β2 and TGF-β3 presenting in the central part of the developing chick retina are essentially required to trigger retinal cell apoptosis, which can create space for incoming axons of retinal ganglion cells to form optic nerve.^298,299^ In mice, however, TGF-β signaling also protects retinal neurons from excessive apoptosis to ensure proper development of eyes.^300^

### Wound healing

Wound healing which happens after tissue injuries generally involves four orderly and overlapping stages known as hemostasis, inflammation, proliferation, and remodeling.^301^ Throughout the healing of cutaneous wounds, all TGF-β isoforms and TβR types are induced in a distinct spatial and temporal pattern.^302,303^ During hemostasis, platelets provide an immediate and abundant supply of TGF-β after wounding, contributing largely to subsequent healing stages by promoting the influx of inflammatory cells and fibroblasts into the wounds due to its chemotactic activity.^302,304–307^ Interestingly, many of the cell types recruited by TGF-β are also active in secreting TGF-β, leading to even higher TGF-β concentrations in the wounds. In ovine skin, all three TGF-β isoforms increase dramatically only one day after wounding, attributed to the expression by epithelial cells, endothelial cells, fibroblasts, and inflammatory cells such as neutrophils, macrophages, and lymphocytes.^302^ During the stage of proliferation and remodeling, TGF-β is implicated in wound re-epithelialization, tissue angiogenesis, and fibroblast activation.^308,309^ Upon cutaneous injury, TGF-β1 is initially expressed by all epidermal keratinocytes adjacent to the wounds but gradually gets excluded from the basal keratinocytes, corresponding to the transient block and subsequent burst of basal keratinocyte proliferation after wounding.^310^ TGF-β1 also contributes to the migration of epithelial sheets at the leading edges of cutaneous wounds through the regulation of integrins and the activation of PI3K.^310–312^ Other TGF-β isoforms such as TGF-β3 can have similar impacts on cell migration during cutaneous wound healing.^313^ As for angiogenesis, TGF-β regulates the proliferation and migration of endothelial cells in vitro and shows potent angiogenic activity when overexpressed or directly applied in vivo.^307,314–321^ A possible mechanism of TGF-β-induced angiogenesis involves the induction of vascular endothelial growth factor (VEGF) in epithelial cells and fibroblasts.^322,323^ Moreover, TGF-β can stimulate fibroblasts to proliferate and produce bioactive factors such as collagen, fibronectin, MMPs, tissue inhibitor of MMPs (TIMPs), and plasminogen activator inhibitor 1 (PAI-1) which contribute to the deposition and remodeling of wound ECM.^304,306,307,315,317,321,324–334^ It can also promote fibroblast-mediated wound contraction through MF differentiation and RHO activation.^335–337^

Apart from the skin, TGF-β also functions in the repair and regeneration of many other tissues. During rat liver regeneration, all TGF-β isoforms are induced in non-parenchymal cells rather than hepatocytes, which however, exhibit upregulation of all TβR types to enhance the responsiveness to TGF-β, which may help to prevent uncontrolled cell proliferation.^338–342^ Similarly, the marked increase in TGF-β and TβR expression following acute pancreatitis suggests the role of TGF-β signaling in pancreatic repair.^343–345^ Upon vascular injury, TGF-β mobilizes MSCs to peripheral blood and further recruits them to the injured sites for vascular repair.^346^ As for cardiac repair after myocardial injury, TGF-β triggers the EndMT of epicardial cells, which then migrate into the injured myocardium to generate various cardiac cell types.^347^ TGF-β also plays a role in cartilage repair by stimulating proteoglycan synthesis in chondrocytes.^348,349^ Moreover, after injury in the nervous system, neurons, astrocytes, microglia, as well as recruited macrophages all upregulate the expression of TGF-β which may contribute to the healing process of the nervous tissues.^350,351^

### Tissue homeostasis

Tissue homeostasis is maintained by the balance between cell proliferation and cell death in which TGF-β acts as a key regulator.

Cell proliferation is generally driven by CDKs through a series of events collectively known as the cell cycle. For most cells, TGF-β inhibits their proliferation, or in other words, triggers their cytostasis by inducing cell cycle arrest in the gap 1 (G1) phase. In epithelial cells and glial cells, TGF-β suppresses the activity of CDKs by activating the transcription of CDK inhibitors (CKIs) such as p15 and p21 to induce cytostasis.^352–355^ The transcriptional activation of CKIs in response to TGF-β is likely mediated by SMADs in cooperation with transcription factor FOXO^355,356^ or specificity protein 1 (SP1).^357,358^ Notably, the SMAD-FOXO complex additionally requires transcription factor C/EBPβ for the induction of p15 but not of p21.^356^ In epithelial cells, TGF-β-mediated upregulation of p15 also prevents the non-inhibitory binding of CKI p27 to CDK4. As a result, p15 and p27 turn to bind their own targets which are respectively CDK4 and CDK2 to exert their inhibitory effects.^359,360^ Interestingly, in murine B cells, TGF-β increases the expression of p27 instead of p21 to trigger cytostasis,^361^ while in human hematopoietic cells, p57 is likely the only TGF-β-induced CKI for cell cycle arrest.^362^ Besides CKIs, TGF-β can also target other proliferative factors such as MYC, inhibitors of DNA binding (IDs), and CDC25A to inhibit cell proliferation as mostly shown in epithelial cells. TGF-β induces the transcriptional repression of MYC through a complex containing SMADs, transcription factors E2F4/5 and C/EBPβ, as well as transcriptional corepressor p107.^356,363,364^ It also inhibits ID1 expression through SMADs which mediate the induction and recruitment of transcriptional repressor activating transcription factor 3 (ATF3) to target ID1 promoter.^365^ As for ID2 which can be induced by MYC at the transcriptional level, its suppression by TGF-β is attributed to the downregulation of MYC or the upregulation of antagonistic MYC repressors known as MYC-associated factor X (MAX) dimerization proteins (MADs).^366,367^ By these means, TGF-β is able to relieve the transcriptional repression on CKIs exerted by MYC and IDs to facilitate the induction of cytostasis.^368–371^ Furthermore, TGF-β can downregulate the activity of the CDK-activating phosphatase CDC25A through several mechanisms such as the transcriptional repression by E2F4-p130-HDAC1 complex,^372^ the inhibitory phosphorylation by RHOA/ROCK1 signaling,^373^ as well as the SMAD3-dependent degradative ubiquitination by E3 ubiquitin ligase complex SCF.^374^ Notably, TGF-β can also stimulate the proliferation of certain cell types, including SMCs, fibroblasts, and chondrocytes, likely due to the induction of autocrine growth factors such as fibroblast growth factor (FGF) and platelet-derived growth factor (PDGF).^324,325,375^

As for cell death, TGF-β can trigger apoptosis which is one of the most common forms of cell death in a wide range of cell types including lymphocytes, hepatocytes, podocytes, glial cells, hematopoietic cells, and epithelial cells. Such effect is generally attributed to SMAD-dependent regulation of B-cell lymphoma-2 (BCL-2) family members. More specifically, TGF-β can upregulate pro-apoptotic BCL-2 family members such as BCL-2-associated X protein (BAX) and BCL-2-interacting mediator of cell death (BIM),^376–379^ meanwhile, it can also downregulate anti-apoptotic BCL-2 family members such as BCL-2 and BCL-extra-large (BCL-XL).^378,380,381^ Apart from BCL-2 family members, many other effectors and pathways are also involved in TGF-β-induced cell apoptosis. A septin-like protein known as apoptosis-related protein in the TGF-β signaling pathway (ARTS) undergoes mitochondrial-to-nuclear translocation to promote cell apoptosis in response to TGF-β.^382^ Death domain-associated protein (DAXX) interacts with TβRII as an intermediary to convey pro-apoptotic TGF-β signal to downstream machinery.^383^ In B cells and hepatocytes, TGF-β triggers the transient activation of TAK1/IKK/NF-κB pathway, sequentially leading to the transcriptional activation of IκB-α, the post-repression of NF-κB, the upregulation of JNK signaling, the increase of activator protein 1 (AP-1) complex activity, and finally, the apoptotic death of cells.^384–386^ In hepatocytes, TGF-β also promotes the expression of growth arrest and DNA damage-inducible β (GADD45β), which functions as a positive mediator of cell apoptosis by acting upstream of p38 MAPK.^387^ As for podocytes, TGF-β can activate both pro-apoptotic p38 signaling and anti-apoptotic PI3K/AKT signaling to regulate their survival and death.^379,388^ In fact, AKT, especially when phosphorylated, can bind to unphosphorylated SMAD3 to inhibit its activity and thus protect several cell types from SMAD-dependent apoptosis. In contrast, TGF-β can prevent the AKT-SMAD3 interaction by triggering SMAD3 phosphorylation to facilitate the cell death program.^389,390^ Moreover, in hematopoietic cells, SMAD-dependent TGF-β signaling induces the expression of a central regulator of phospholipid metabolism known as SRC homology 2 (SH2) domain-containing inositol 5’-phosphatase (SHIP) to inhibit AKT phosphorylation as well as cell survival.^391^ Furthermore, TGF-β triggers the apoptosis of oligodendrocytes and epithelial cells by inducing transcription factors TGF-β-inducible early genes (TIEGs) to downregulate BCL-XL expression.^392–394^ Notably, TGF-β is also found to promote cell survival in certain cases.^300,395–398^ Related mechanisms involve the AKT-dependent inhibition of FOXO3 as in epithelial cells,^399^ the suppression of AKT and the induction of BCL-2 as in pre-B lymphocytes,^400^ the early induction and phosphorylation of c-Jun and consequential attenuation of JNK as in lung carcinoma cells,^401^ the downregulation of CD95L and p53 as well as the upregulation of NF-κB, BCL-XL, and p21 as in HSCs.^402^

### Immune homeostasis

Generally, TGF-β functions to suppress the activity of multiple immunocompetent cells while inducing the phenotypes of several immune immunosuppressive cells. For this reason, it is regarded as one of the most potent immunosuppressive cytokines which are of vital importance to the maintenance of immune homeostasis and self-immune tolerance.^403^

#### Cytotoxic T lymphocytes (CTLs), T helper type 1 (Th1), and Th2 cells

TGF-β prevents naïve T cells from differentiating into classical effecter T cells through numerous mechanisms. For CD8+ T cells which can develop into CTLs upon activation, TGF-β inhibits their functions by suppressing the expression of cytolytic factors such as perforin, granzyme A, granzyme B, Fas ligand, and interferon (IFN)-γ. Mechanically, the encoding genes of granzyme B and IFN-γ are directly recognized by SMADs and transcription factor ATF1 which both bind to the gene promoter regions to mediate transcriptional repression in response to TGF-β signaling.^404^ The suppression of IFN-γ release is also correlated to the reduction of transcription factor T-box expressed in T cells (T-BET)^405^ while the decrease in Fas ligand expression is partially attributed to the downregulation of MYC.^406^ In CD4+ T cells, TGF-β inhibits the phosphorylation of T-cell kinase (ITK) to decrease the influx of calcium ion and subsequent activation of nuclear factor of activated T cells (NFATC) which are both critical events for Th1 and Th2 cell differentiation.^407^ TGF-β also suppresses the expression of transcription factors T-BET and GATA-3 in CD4+ T cells which act as master transcriptional activators during Th1 and Th2 cell development respectively.^408–410^

#### Tregs, Th9, and Th17 cells

TGF-β induces the expression of transcription factor forkhead box P3 (FOXP3) in an interleukin (IL)-2-dependent manner in CD4+ CD25− naïve T cells to convert them into CD4+ CD25+ Tregs which can express TGF-β and inhibit other T cell proliferation with potent immunosuppressive activity.^411–414^ Similarly, TGF-β can induce the generation of Tregs from CD8+ T cells through the expression of FOXP3.^415,416^ Interestingly, IL-4 inhibits the induction of FOXP3 by TGF-β in naïve CD4+ T cells, instead, both cytokines cooperate to drive the differentiation of another Th cell subset known as Th9 cells by inducing the expression of transcription factor purine-rich box-1 (PU.1).^417–419^ Unlike the immunosuppressive Tregs, these IL-9- and IL-10-secreting cells can potently promote tissue inflammation.^417–420^ In addition, inflammatory cytokines such as IL-1β, IL-6, IL-21, and IL-23 also suppress TGF-β-induced FOXP3 in naïve CD4+ T cells, meanwhile, they elevate the activity of a TGF-β-induced transcription factor known as retinoic acid receptor-related orphan receptor γt (RORγt) to contribute to the generation of Th17 cells. This pro-inflammatory Th cell subset characterized by IL-17 expression plays important roles in anti-microbial defense and autoimmunity.^421,422^

#### B cells

As critical effectors of humoral immune responses, B cells mainly function by secreting antibodies which are also known as immunoglobulins (Igs). TGF-β decreases B cell Ig secretion by inhibiting the synthesis and the switch from the membrane form to the secreted form of Ig messenger ribonucleic acids (mRNAs).^423^ More specifically, TGF-β selectively inhibits the expression of Ig λ light chains while inducing less pronounced reductions in Ig κ light chains,^423,424^ moreover, it suppresses the production of isotypes IgM and IgG but enhances the class switching to isotype IgA.^423,425,426^ Notably, TGF-β-induced IgA with poor specificity is considered insufficient to mediate immune responses such as antibody-dependent cellular cytotoxicity (ADCC) and antibody-dependent cellular phagocytosis (ADCP).^427,428^ Furthermore, TGF-β can convert B cells into regulatory B cells (Bregs) which produce numerous factors such as TGF-β, IL-10, IL-35, Fas-L, and programmed death-ligand 1 (PD-L1) to mediate immunosuppression.^429–432^

#### Natural killer (NK) cells

NK cells are cytotoxic lymphocytes of the innate immunity. TGF-β suppresses NK cell development by downregulating transcription factor E4 promoter-binding protein 4 (E4BP4) in a SMAD3-dependent manner.^433^ The SMAD3 also decreases NK cell IFN-γ secretion through the inhibition of E4BP4 and T-BET.^433,434^ Moreover, TGF-β downregulates the surface expression of NK triggering receptors such as NKP30 and NK group 2 member D (NKG2D) which are responsible for the recognition and killing of target cells.^435,436^ It also negatively regulates the expression of cytolytic factors such as granzyme A, granzyme B, and perforin through SMAD signaling to further impair NK cytotoxicity.^434,436^

#### DCs, macrophages, and neutrophils

DCs, macrophages, and neutrophils can function as antigen-presenting cells (APCs), which are the keys to the activation of adaptive immune responses. TGF-β can impair antigen presentation through the downregulation of major histocompatibility complex (MHC) molecules.^437–439^ It also reduces the expression of IL-12 and co-stimulatory molecules such as CD40 in macrophages and CD80, CD83, and CD86 in DCs to interfere in APC-mediated immune cell activation.^440,441^ Apart from antigen presentation, TGF-β also inhibits the cytotoxicity of macrophages, on one hand, through the downregulation of cytotoxic factors, such as TNF-α and nitric oxide (NO),^442–446^ on the other hand, by suppressing the activity of Fcγ receptors (FcγRs) which function to mediate the ADCC and ADCP of macrophages.^447^ Moreover, TGF-β can trigger the polarization of macrophages and neutrophils from classical M1 macrophages and N1 neutrophils to alternative M2 macrophages and N2 neutrophils which are characterized by multiple immunosuppressive properties.^439,448–450^

## TGF-β signaling in disease

**Fig. 6:**
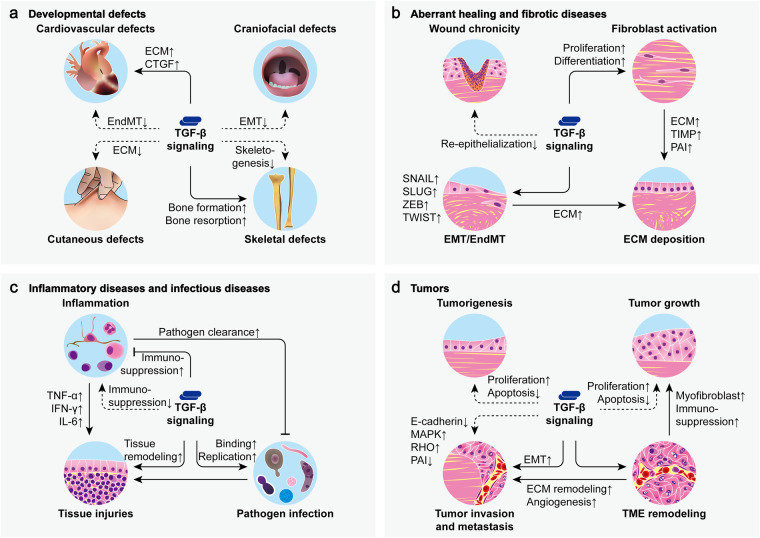
TGF-β signaling in disease. Dysfunctional TGF-β signaling is involved in numerous pathological processes. **a** Mutations that lead to decreased or increased TGF-β signaling can cause various developmental defects. **b** Deficient TGF-β signaling contributes to wound chronicity while excess TGF-β signaling leads to wound scarring and tissue fibrosis by stimulating ECM deposition through fibroblast activation and EMT/EndMT. **c** Dysfunctional TGF-β signaling exacerbates tissue injuries in inflammatory diseases and infectious diseases by promoting inflammation, pathogen infection, and tissue remodeling. **d** Aberrant TGF-β signaling is implicated in all aspects of tumor development including tumorigenesis, tumor growth, tumor invasion, tumor metastasis, as well as tumor microenvironment (TME) remodeling. (CTGF, connective tissue growth factor; IFN-γ, interferon-γ; IL-6, interleukin-6; solid arrows from TGF-β indicate excessive TGF-β signaling, dashed arrows from TGF-β indicate deficient TGF-β signaling)

Dysfunctional TGF-β signaling can play key roles in numerous pathological processes, contributing to the disorders of developmental defects, aberrant healing, fibrotic diseases, inflammatory diseases, infectious diseases, as well as tumors (Fig. 6).

### Developmental defects

Loss of TβRI or TβRII functions due to homozygous mutations generally results in embryonic lethality in mice due to defects in the hematopoiesis and vasculogenesis of yolk sac.^451,452^ However, the lack of different TGF-β isoforms can lead to distinct phenotypes in mice, consistent with the isoform-specific roles of TGF-β in embryonic development. TGF-β1-knockout mice show no gross developmental abnormalities in spite of the defective hematopoiesis and vasculogenesis in yolk sac during embryonic development.^452–454^ In contrast, TGF-β2-knockout mice exhibit perinatal mortality and a wide range of developmental defects in heart, lungs, bones, eyes, inner ears, craniofacial structures, urogenital organs, and hair follicles.^290,455–458^ TGF-β3-knockout mice also die shortly after birth but show no detectable abnormalities except for cleft palate and abnormal lung development.^459,460^ Notably, palatal shelves that fail to elevate in TGF-β2-knockout mice undergo elevation in TGF-β3-knockout mice but still fail in fusion.^455,459,460^ Also, branching morphogenesis and respiratory epithelial cell differentiation which appear normal in the lungs of TGF-β2-knockout mice are defective in TGF-β3-knockout mice.^455,459^ In humans, loss-of-function mutations of a single TGF-β signaling component such as TGF-β2,^461–463^ TGF-β3,^464–466^ TβRI,^467–469^ TβRII,^470–472^ SMAD2,^473–475^ or SMAD3^476–478^ can cause Loeys-Dietz syndrome (LDS), an autosomal dominant connective tissue disorder with a range of cardiovascular, skeletal, craniofacial, and cutaneous manifestations. LDS patients typically present with features including congenital heart defects, aneurysms, arterial tortuosity and dissections, skeletal overgrowth, cervical spine instability, clubfoot deformity, craniosynostosis, hypertelorism, bifid uvula, cleft palate, thin skin, and mental retardation. Dermal fibroblasts derived from LDS patients demonstrate impaired deposition of extracellular collagen and elastin, suggesting a possible mechanism of the connective tissue defects of the patients.^479,480^ However, the aortic tissues of LDS patients show increased accumulation of collagen, elevated expression of connective tissue growth factor (CTGF), and enhanced activity of non-mutant TGF-β signaling components.^461–463,465,467,468,475,476,481,482^ Therefore, primary downregulation and compensatory upregulation of TGF-β signaling are both responsible for the abnormalities of LDS.

Excessive TGF-β signaling can also act as a primary pathogenic factor in developmental defects. In mice, overexpression of TGF-β or SMAD can lead to developmental abnormalities in several tissues, such as skin,^483,484^ bones,^485^ eyes,^486^ lungs,^487,488^ mammary glands,^489–491^ salivary glands,^492^ and central nervous system.^493^ In humans, Camurati-Engelmann disease (CED), a progressive bone dysplasia inherited in an autosomal dominant manner, is ascribed to mutations of TGF-β1, which lead to increased TGF-β1 activation and signaling.^494,495^ This disease is characterized by hyperostosis and sclerosis of the long bones and the skull.^496,497^ Studies on CED have suggested that hyperactive TGF-β1 in the bone microenvironment can induce osteoclasts and osteoblasts to increase but cluster in separated areas, uncoupling bone resorption and formation to cause bone remodeling defects.^494,498,499^

### Aberrant healing and fibrotic diseases

Dysregulated TGF-β signaling can contribute to the tissue damage in aberrant healing and fibrotic diseases which are caused by all kinds of injuries such as wounding, burns, radiation, infection, and inflammation.

#### Aberrant healing

The lack of TGF-β and TβR expression is commonly found in the chronic wounds in patients, indicating that deficient TGF-β signaling may lead to wound chronicity and even unhealing.^500–505^ However, in vivo studies in mice have reported quite complicated findings. An activating mutation of TβRI can lead to a regenerative healing phenotype which enables rapid regeneration of normal tissues with differentiated structures instead of scar formation in ear punch wounds.^506^ Paradoxically, overexpression of TGF-β1 in keratinocytes accelerates the re-epithelialization in partial-thickness cutaneous wounds but slows that of full-thickness cutaneous wounds.^507,508^ In TGF-β1-deficient mice, the healing of full-thickness cutaneous wounds is initially normal but ultimately damaged by severe inflammatory diseases.^509^ In immunodeficient mice without inflammatory diseases, the lack of TGF-β1 still leads to significant delays in each healing stage of full-thickness cutaneous wounds.^510^ However, loss of TGF-β signaling in keratinocytes due to expression of dominant negative TβRII leads to increased proliferation and reduced apoptosis, thus facilitating the re-epithelialization in full-thickness cutaneous wounds.^511^ Furthermore, cutaneous wound healing is accelerated in mice lacking SMAD3 but is aberrant in mice lacking SMAD4 exclusively in keratinocytes.^512,513^

In contrast to chronic wounds, hypertrophic scars and keloids both characterized by overabundant ECM deposition are the results of hyperactive cutaneous wound healing. In fact, the expression of TGF-β and TβR which decreases eventually in normal cutaneous wounds remains elevated in hypertrophic scars and keloids.^514–518^ In contrast to normal cutaneous fibroblasts, both keloid fibroblasts and hypertrophic scar fibroblasts are significantly higher in collagen production, however, only keloid fibroblasts exhibit increased sensitivity to TGF-β stimulation.^519^ For keloid fibroblasts, overexpressed TGF-β can promote the resistance to apoptosis, the ability of proliferation, the conversion to MFs, and the expression of CTGF and VEGF, thus contributing to the ECM deposition, focal adhesion, fibrous growth, and angiogenesis in keloid tissues.^518,520–523^

#### Fibrotic diseases

Besides wounding, other forms of injurious stimulation can also cause excessive ECM deposition in different kinds of tissues, leading to fibrotic diseases, which are closely associated with the hyperactivity of TGF-β signaling.

TGF-β expression is significantly elevated in fibrotic lungs in various cases such as idiopathic pulmonary fibrosis (IPF) and cystic fibrosis (CF).^524–528^ In situ hybridization and immunohistochemical staining suggest that alveolar macrophages and epithelial cells are likely the major sources of TGF-β which contribute to the fibrosis of lungs.^526–528^ In vitro studies show that TGF-β1 can trigger the EMT of alveolar epithelial cells and enhance the activity of lung fibroblasts to mediate fibrogenic effects.^529–532^ Transgenic expression of TGF-β1 in murine and rat lungs induces pulmonary fibrosis which is accompanied by alveolar EMT, MF differentiation, and mononuclear-rich inflammation.^532–535^ Interestingly, the suppression of TGF-β1, the deletion of TβRII, the ablation of SMAD3, the upregulation of SMAD7, but the administration of TGF-β3 can all significantly protect mice from experimentally induced pulmonary fibrosis.^535–539^

Similarly, the fibrotic kidneys of human glomerulonephritis, IgA nephropathy, diabetic nephropathy, lupus nephritis, as well as renal allografts in chronic rejection all show significant increases in three TGF-β isoforms in the glomeruli and tubulointerstitium where ECM deposition and PAI-1 production is closely related to the expression of TGF-β1 isoform in particular.^540–543^ In vitro, TGF-β1 stimulates kidney fibroblasts, mesangial cells, glomerular epithelial cells, and tubular epithelial cells to produce several ECM components and remodelers such as collagen, fibronectin, laminin, proteoglycan, MMP, and TIMP.^544–550^ TGF-β1 also contributes to the EMT induction and MF differentiation in renal fibrosis.^550^ Transgenic mice that have increased levels of TGF-β1 in plasma develop progressive renal disease characterized by glomerulosclerosis and tubulointerstitial fibrosis with TIMP overexpression and ECM deposition in sub-endothelial and mesangial locations.^551,552^

In fibrotic livers, TGF-β1 expression increases markedly with fibrogenic activity.^553–556^ Induction of TGF-β1 expression in murine livers leads to hepatic fibrosis characterized by prominent ECM deposition in peri-sinusoidal areas with activation of HSCs and apoptosis of hepatocytes.^557,558^ Notably, activated HSCs which play a major role in hepatic fibrosis can provide an important source of TGF-β,^559^ while overproduced TGF-β can in turn activate several signaling pathways such as those of SMAD, MEK, JNK, PI3K, and JAK/STAT in HSCs to contribute to their functions.^236,237^

As for the cardiovascular system, TGF-β is also elevated during myocardial fibrosis, valve fibrosis, and arteriosclerosis, generally attributed to the expression by SMCs, fibroblasts, endothelial cells, and inflammatory cells such as macrophages.^560–569^ On one hand, TGF-β can stimulate cardiovascular fibroblasts to differentiate into MFs and produce ECM components and remodelers,^562,563,570–573^ on the other hand, it can also stimulate endothelial cells to undergo EndMT to induce their fibrogenic phenotype.^569,574,575^

Furthermore, TGF-β is widely involved in the fibrosis of many other tissues and diseases as in the cases of cutaneous fibrosis,^576,577^ muscular fibrosis,^578^ pancreatic fibrosis,^579–582^ myelofibrosis,^583,584^ adenomyosis,^585^ autoimmune diseases,^238,527,573,586–589^ and infectious diseases.^590–593^

### Inflammatory diseases and infectious diseases

Inflammatory diseases and infectious diseases can demonstrate aberrant immune responses and various tissue injuries which usually implicate the dysfunction of TGF-β signaling.

#### Inflammatory diseases

Since TGF-β acts as a negative regulator to maintain immune homeostasis, deficient TGF-β signaling can lead to hyperactive immune responses, contributing to the pathology of numerous inflammatory diseases. TGF-β1-null mice initially appear normal after birth but soon develop a rapid wasting syndrome accompanied by a multifocal inflammatory disease which leads to organ failure and early death by 3-4 weeks of age.^453,594–596^ Many organs in these mice, including heart, lungs, stomach, liver, pancreas, and muscles, all exhibit massive infiltration of inflammatory cells such as lymphocytes, macrophages, and granulocytes. Moreover, their total numbers of blood leukocytes increase mainly due to the elevated absolute numbers of neutrophils and monocytes, while their levels of autoantibodies, MHC molecules, and inflammatory cytokines such as IFN-γ, TNF-α, and CCL3 also rise correspondingly in serum or tissues.

In the absence of any pathogens, the inflammatory diseases in TGF-β1-knockout mice actually resemble a special group of inflammatory diseases known as autoimmune diseases, which are characterized by dysregulated immune responses attacking self-tissues. In fact, even cell type-specific loss of TGF-β signaling can lead to the development of various autoimmune diseases in mice.^597–604^ In patients with autoimmune diseases such as systemic lupus erythematosus (SLE),^605–607^ systemic sclerosis (SSc),^608–611^ rheumatoid arthritis (RA),^612–614^ Sjögren’s syndrome,^586,614–616^ Crohn’s disease,^587,617–619^ ulcerative colitis (UC),^617–622^ autoimmune hepatitis (AIH),^623,624^ and Hashimoto’s thyroiditis (HT),^606,625,626^ the levels of TGF-β or TβR in tissues or circulation are associated with the presence, activity, and severity of the diseases. Notably, although all these diseases show correlations with dysregulated TGF-β signaling, their correlations with TGF-β levels can be either positive or negative. Some cases of the diseases are likely caused by insufficient TGF-β expression and thus exhibit decreased TGF-β production.^619,622,627–630^ In other cases, however, the autoimmune inflammation is likely attributed to impaired cell responsiveness to TGF-β especially due to deficient TβR functions, therefore, TGF-β production is elevated as a compensatory response.^624,628,631–635^

Allergic diseases, including asthma, allergic rhinitis, food allergy, and atopic dermatitis, are another group of inflammatory diseases that are caused by aberrant immune responses to harmless environmental antigens. TGF-β production is increased in the airways and serum of asthmatic patients and is further increased after allergen exposure, disease progression, or certain treatments.^636–646^ Bronchial epithelial cells, fibroblasts, SMCs, eosinophils, neutrophils, and macrophages can all contribute to the excessive TGF-β production in asthmatic patients.^641–650^ However, the functions of TGF-β are seemingly contradictory in the context of allergic airway inflammation, for TGF-β can either enhance or suppress the activity of eosinophils, lymphocytes, macrophages, and mast cells in asthma.^648,651–661^ Nevertheless, it is clear that TGF-β can promote asthmatic airway remodeling by inducing airway EMT,^662,663^ ECM production,^649,650^ MF differentiation,^664,665^ and smooth muscle hyperplasia.^647^ In patients with allergic rhinitis, TGF-β levels in serum are found dependent on allergen exposure, while TGF-β and TβR expression in nasal mucosa is noticed correlated with intra-epithelial mast cell abundance.^666–668^ In fact, allergen challenge can activate TGF-β signaling in the mast cells and epithelial cells in nasal mucosa which may contribute to the mast cell accumulation and goblet cell hyperplasia in allergic rhinitis.^669,670^ Allergen challenge can also induce the loss of TGF-β1-expressing Bregs and Tregs which function to suppress the inflammatory Th2 responses of allergic rhinitis. However, with prolonged challenging time, the proportion of TGF-β1-expressing Bregs and Tregs can gradually recover to reconstitute the immune homeostasis in nasal mucosa.^671^ Similarly, TGF-β can inhibit the Th2 responses of food allergy by promoting Treg activity in the intestines.^603,672,673^ Therefore, reduced TGF-β1 expression in the intestinal epithelial cells and mononuclear cells of patients with food allergy can partially account for the development of the disease.^603,674^ Moreover, TGF-β can inhibit the pathology of atopic dermatitis by suppressing B cell maturation, mast cell activation, eosinophil infiltration, as well as the secretion of IgE, TNF-α, and histamine by those cells.^675–677^ Aberrant TGF-β expression or attenuated cell responsiveness discovered in patients with atopic dermatitis may play a key role in the disorder.^678–680^

Furthermore, TGF-β signaling is implicated in the pathology of other inflammatory diseases and inflammation-related diseases such as bronchitis,^642^ pancreatitis,^681–683^ glomerulonephritis,^684,685^ osteomyelitis,^686^ arthritis,^687^ diabetes,^688^ and Alzheimer’s disease (AD).^689,690^

#### Infectious diseases

Infectious diseases caused by different kinds of pathogenic organisms can result in tissue damage due to diverse pathogen virulence and dysregulated host responses.

TGF-β can function to reduce pathogen burdens as well as tissue injuries in some cases of infection. In patients with H1N1 influenza A virus sepsis, blood TGF-β levels are negatively correlated with clinical severity scores on admission.^691^ Consistently, increased TGF-β activity in mice confers resistance against lethal influenza infection due to reductions in both viral titers and pulmonary inflammation.^692,693^ TGF-β expression also prevents mice from coxsackievirus-induced myocarditis and type 1 diabetes in a Treg-dependent manner.^694,695^ Moreover, TGF-β acts as a pro-survival factor to protect murine neurons and intestinal epithelial cells against cell death during reovirus infection.^696,697^ As for bacterial infection, TGF-β can attenuate sepsis-induced tissue injuries through mechanisms involving the induction of Tregs.^698^ It also enhances the pathogen clearance and host resistance of mice during the infection of *Streptococcus pneumoniae,*^699^ *Streptococcus pyogenes,*^700^ *Listeria monocytogenes,*^701^ and *Yersinia enterocolitica,*^702^ likely, by suppressing IFN-γ, TNF-α, and IL-6 production while promoting Th17 and Treg responses. In rats with pulmonary cryptococcosis, TGF-β reduces fungal burdens by promoting the lysozyme secretion of macrophages, meanwhile, it also limits inflammation by inhibiting macrophage phagocytosis, chemokine production, and oxidative burst.^703^ Moreover, TGF-β can be protective during parasitic infection. The lack of TGF-β exacerbates the severity of murine malaria infection, whereas TGF-β treatment, in contrast, suppresses plasmodium proliferation and prolongs mice survival with decreased TNF-α and increased IL-10 in serum.^704^ During *Trypanosoma congolense* infection, exogenous TGF-β1 confers early protection against parasitemia, anemia, splenomegaly, and mortality due to enhanced macrophage activity and Th1 responses which are characterized by increased NO, IFN-γ, TNF-α, IL-12, and IgG2a production.^705^ During Toxoplasma infection, TGF-β can prevent tissue damage by reducing inflammatory cell infiltration and cytokine production, while it can also improve the outcomes of infection-related abnormal pregnancy by promoting Treg functions and suppressing NK cytotoxicity.^706–709^ Furthermore, TGF-β can prevent the lung injuries during hookworm infection by inducing the immunosuppressive activity of myeloid cells to reduce Th2 responses.^710^

In other cases of infection, however, TGF-β can turn to facilitate pathogen infection and tissue injuries. In clinical patients, circulating TGF-β1 levels are positively correlated with the severity and mortality of severe community-acquired pneumonia (CAP)^711^ and sepsis-induced acute respiratory distress syndrome (ARDS).^712^ Increased TGF-β production can impair the anti-bacterial functions of neutrophils, uncouple the cytokine production and glycolysis of macrophages, and suppress the IL-2 expression and proliferation of T cells to participate in the pathology of sepsis.^713–715^ As for bacterial infection in local tissues, on one hand, TGF-β can upregulate fibronectin and integrins in hosts to promote bacterial adhesion and invasion,^716,717^ on the other hand, it can attenuate anti-infectious innate responses and Th1 responses while inducing immunotolerant Treg responses to facilitate the immune escape of the pathogens.^718–722^ TGF-β-mediated immunosuppression can also contribute to viral infection, as elevated TGF-β expression during viral infection not only impairs early innate immunity such as IFN responses, NK functions, and macrophage activity but also suppresses the adaptive immune responses of T cells and B cells.^428,723–731^ Notably, TGF-β can also enhance viral infection through certain pathogen-specific mechanisms as in the cases of human immunodeficiency virus type 1 (HIV-1) infection,^732–734^ human T-cell leukemia virus type I (HTLV-I) infection,^735^ hepatitis C virus (HCV) infection,^736^ Zika virus (ZIKV) infection,^737^ as well as rubella virus (RuV) infection.^738^ Furthermore, TGF-β can promote the survival and growth of parasites in hosts through downregulation of NO, IFN-γ, TNF-α, IL-6, IL-17, and Th17 cells as well as upregulation of IL-4, IL-10, and Treg cells, contributing to the infection of *Fasciola hepatica,*^739^ *Echinococcus multilocularis,*^740^ *Toxoplasma gondii,*^741^ Leishmania,^742^ and Plasmodium.^743,744^

### Tumors

It is generally accepted that TGF-β acts as a tumor suppressor during the early stages of tumorigenesis but turns into a tumor promotor at later stages of tumor development.

#### Tumorigenesis

Evidence from animal models firmly establishes the suppressor role of TGF-β signaling in early tumorigenesis. TGF-β and its receptors can be strongly induced in the murine epidermis upon exposure to carcinogens that tend to disrupt tissue homeostasis and cause oncogenic transformation.^745,746^ Increased TGF-β expression in murine epidermis can potently attenuate cell proliferation and confer resistance to hyperproliferation induced by carcinogens.^316,746,747^ Similarly, in murine mammary epithelia, the overexpression of TGF-β or TβR can result in remarkable protection from carcinogen-induced tumorigenesis with reduced premalignant lesions, prolonged tumor latency, and decreased cancer incidence.^25,491,748–750^ Such tumor-inhibitory effects by TGF-β signaling are attributed to the early apoptosis of differentiating cells and, more importantly, the premature senescence of stem cells which reduces the reproductive capacity of the mammary epithelia and thus decreases the frequency with which transforming mutations may occur and be fixed in the cell population.^491,748^

In contrast, loss of TGF-β signaling can be an early event that contributes to tumorigenesis. In clinical patients, heterogeneous patterns of TβRII expression in normal breast lobular units as well as loss of TβRII expression in breast epithelial hyperplastic lesions are both associated with increased risks of invasive breast cancer.^751^ More convincing evidence is provided by germline mutations of TGF-β signaling components which show strong correlations with increased risks of tumorigenesis. Loss-of-function TβRI mutations can result in an autosomal dominant skin cancer condition known as multiple self-healing squamous epithelioma (MSSE) or Ferguson-Smith disease (FSD) which is characterized by multiple squamous-carcinoma-like skin tumors that invade locally and then regress spontaneously after several months.^752,753^ Inactivating TβRII mutations are considered causative of some cases of hereditary nonpolyposis colorectal cancer (HNPCC) or Lynch syndrome, an autosomal dominant cancer predisposition syndrome, by impairing cell growth inhibition in response to TGF-β.^754^ Moreover, germline mutations of SMAD4 are responsible for juvenile polyposis, an autosomal dominant syndrome predisposing to gastrointestinal hamartomatous polyps and cancers.^755,756^ Mechanically, impaired TGF-β signaling can cause serious disturbance to tissue homeostasis, thus largely facilitating the development of pre-neoplastic lesions, as well as subsequent tumors, as shown in different murine tissues with deficiencies in the activity of TGF-β,^757,758^ TβR,^749,759–770^ or SMAD.^657,771–776^ Among them, TβR-deleted murine epithelia exhibit significant reductions in p15 and p21 and remarkable increases in MYC expression and RAS/ERK signaling, accompanied by elevated cell proliferation, reduced cell apoptosis, and enhanced cell malignant transformation to become tumorigenic.^762–765^

Furthermore, TGF-β can provide additional protection against tumorigenesis by controlling pathogen infection,^777^ inhibiting excessive inflammation,^778–781^ reducing genomic instability,^782^ inducing replicative senescence,^783^ and regulating epithelial-mesenchymal interaction.^784^

#### Tumor growth

TGF-β can inhibit tumor growth by triggering cytostasis and apoptosis through similar mechanisms as it does in cells from normal tissues. In tumor cells, TGF-β signaling induces cell cycle arrest by targeting effectors, such as p15,^354,356^ p21,^355,785,786^ p27,^361^ MYC,^363^ ID,^787^ and CDC25A,^374,786^ while it also induces apoptotic cell death through effectors including CTGF,^788^ programmed cell death 4 (PDCD4),^789^ Fas receptor,^790^ death-associated protein kinase (DAPK),^791^ DAXX,^383^ IκB-α,^384,386^ sex-determining region Y (SRY)-box 4 (SOX4),^792^ ARTS,^382^ TIEGs,^793^ as well as several BCL-2 family members.^794–800^ Consistently, primary tumors induced from murine tissues with intact TGF-β signaling pathways are initially responsive to TGF-β-mediated inhibitory effects.^491,749,759,764,801,802^

On the contrary, deficient TGF-β signaling can potently promote the growth of tumors. The downregulation of tumor TGF-β signaling in many cases is attributed to reduced expression or inactivating mutations of TβR or SMAD, as shown in various tumor types such as leukemia,^772^ lymphoma,^803,804^ esophageal cancer,^805–807^ gastric cancer,^808^ colorectal cancer,^30,807,809–811^ pancreatic cancer,^32,812,813^ biliary cancer,^812^ ampullary cancer,^814^ thyroid cancer,^815^ prostate cancer,^816,817^ breast cancer,^818^ ovarian cancer,^819^ endometrial cancer,^808^ genital squamous cell carcinomas (SCC),^764^ head and neck SCC,^820–823^ etc. These changes are able to confer resistance to the tumor-inhibitory effects of TGF-β. In mouse models, tumors developed from tissues with deletion or inactivation of TβR exhibit increased cell proliferation and decreased cell apoptosis, accompanied by reduction in p15, p21, and p27, the elevation of MYC, cyclin D1, and epidermal growth factor receptor (EGFR), as well as activation of STAT3 and PI3K/AKT pathways.^761–765^ Interestingly, reconstituted expression of TβRII in tumor cells with corresponding deficiency not only restores the inhibitory responses to TGF-β but also significantly attenuates the tumorigenicity of these cells.^824^

Notably, TGF-β can fail to suppress the growth of tumors where there is likely no loss of functional TGF-β signaling components, and even formerly inhibited tumor cells can subsequently resume proliferating in vitro and develop larger tumor masses in vivo.^354,825,826^ On one hand, such resistance may result from the dysfunction of the downstream targets of TGF-β signaling such as CKIs.^354^ On the other hand, the tumor-suppressive signaling of TGF-β can be offset or interfered by enhanced I-SMAD activity^827^ or potent oncogenic factors such as E1A,^828,829^ EVI1,^148,830^ SKI,^150,152^ SNO,^153^ MYC,^831^ ID2,^832^ mutant p53,^833^ as well as RAS/RAF/ERK signaling.^162,834^ Moreover, TGF-β-mediated tumor-promoting effects can also account for the enhanced tumor growth in vivo, as discussed in a later section.

#### Tumor invasion and metastasis

Contrary to its role as a suppressor of tumor growth, TGF-β generally acts as a promoter of tumor invasion and metastasis especially in advanced tumors. Upregulation of TGF-β as well as its receptors is associated with disease progression and poor prognosis in some patients with tumors such as breast cancer,^835,836^ pancreatic cancer,^837,838^ and gastric cancer.^839^ Consistently, TGF-β overexpression or pre-treatment enables tumor cells to form increased metastases in vivo,^825,840^ while loss of TGF-β responsiveness due to the introduction of dominant negative TβRII decreases the metastatic efficiency of high-grade tumor cells.^841,842^ Moreover, tumors derived from transgenic murine epithelia that overexpress TGF-β or TβR are significantly more malignant and more invasive.^491,749,750,802,843^ Notably, these TGF-β-overexpressing tumor cells are more likely to undergo the transition from epithelial cell phenotype into spindle cell phenotype which is the most malignant and invasive cell type.^802,843^ This indicates that TGF-β can facilitate the progression of epithelial-derived tumors through the induction of EMT which is inoperative in tumors with deficiencies in TβR or SMAD.^761,770,774,842,843^ Similar to the EMT of normal cells, TGF-β-induced EMT of tumor cells is characterized by changes in keratin, integrin, cadherin, catenin, claudin, vimentin, occludin, fibronectin, and MMP expression which can contribute to the invasive and metastatic capacity of tumors.^197,198,200,203,230,232,750,774,802,843–848^

However, loss of functional TGF-β signaling components can occur in tumor cells during disease progression.^759,809^ In fact, reduced TGF-β signaling can also contribute to tumor invasion and metastasis. For some patients, decreased expression of TβR is correlated with higher tumor grades, later clinical stages, and worse clinical prognosis.^805,816,818^ A large number of cell models and mouse models also demonstrate that tumors lacking TGF-β signaling tend to be more malignant and more aggressive.^758,760–762,764,801,841,843,849–852^ Relevant mechanisms in these cases involve the loss of E-cadherin,^761^ the reduction in PAI,^849^ the increase in RHO/RAC signaling,^843^ the activation of integrin/focal adhesion kinase (FAK)/SRC/MAPK pathway,^764^ and more importantly, the overexpression of various pro-invasive and pro-metastatic factors. In mouse models, deficient TGF-β signaling can stimulate tumor cells and stromal cells to produce high levels of TGF-β and other tumor-promoting factors such as CTGF, VEGF, IL-1β, C-X-C motif chemokine ligand (CXCL8), CXCL12, cyclooxygenase(COX)-2, MMPs, collagen, and tenascin C (TNC) which can strongly promote tumor angiogenesis, fibroblasts activation, immune infiltration, and ECM remodeling.^760–764,774,843,850^

#### Tumor microenvironment (TME) remodeling

TGF-β can stimulate tumor progression even when its signaling pathways are unavailable in the tumor cells, indicating its additional tumor-promoting effects exerted on tumor stroma.^760–764,843^ Fibroblasts, endothelial cells, and immune cells are the major stromal cell types in TME and can all be manipulated by TGF-β in favor of tumor progression.

Actively produced TGF-β in the TME can stimulate the chemotactic migration of fibroblasts and convert them into MFs which are also known as cancer-associated fibroblasts (CAFs) in terms of tumors.^305,853^ Activated CAFs can in turn repay TME with more TGF-β as well as other tumor-promoting factors such as TGF-α, FGF, HGF, PDGF, and CTGF to exert a strong stimulation on tumor growth.^324,853–857^ Moreover, TGF-β regulates the production of various ECM components and remodelers by CAFs to facilitate the migration of tumor cells during invasion and metastasis.^855,858^ Interestingly, fibroblasts with the loss of TβRII can also contribute to tumor development through the production of TGF-α, HGF, and macrophage-stimulating protein (MSP).^859^

Endothelial cells can also be converted into CAFs through TGF-β-mediated EndMT.^860^ More importantly, TGF-β promotes the angiogenesis of endothelial cells by inducing VEGF production in tumor cells and fibroblasts.^323,354,750,861,862^ TGF-β also disrupts inter-endothelial junctions to increase the vascular permeability in TME through the process of EndMT and the induction of angiopoietin-like 4 (ANGPTL4).^863^ Therefore, TGF-β-mediated angiogenesis not only increases the blood supply to tumors to favor their growth but also provides tumors with more accessible entrances into the circulation to form metastasis.

Furthermore, TGF-β can modulate immune cell activity to facilitate tumor survival and development. TGF-β inhibits the tumoricidal activity of macrophages and neutrophils and polarizes them into tumor-promoting M2 macrophages and N2 neutrophils, which are also known as tumor-associated macrophages (TAMs) and tumor-associated neutrophils (TANs) in terms of tumors.^439,442,444,448,449,864^ It also promotes the functions of Tregs while suppressing the cytotoxicity of CTLs and NK cells to facilitate tumor evasion from immune surveillance.^865–869^ Moreover, TGF-β can inhibit the expression of MHC antigens in tumor cells to further attenuate their recognition by adaptive anti-tumor immunity.^870,871^ However, TGF-β-mediated downregulation of MHC antigens and NKG2D ligands can increase tumor susceptibility to NK cytotoxicity to some extent.^233,872^

## TGF-β-targeting therapies

To rectify the dysfunction of TGF-β in different kinds of diseases, several targeted therapies have been developed to regulate TGF-β activity at the levels of biosynthesis, activation, and signaling. Many completed clinical trials have preliminarily confirmed the safety and efficacy of some therapeutic strategies, while there are still numerous clinical trials ongoing at present (Table 1).

**Table 1:** Ongoing clinical trials of TGF-β-targeting therapies

Targets	Strategies	Treatments	Diseases	Clinical trials
TGF-β2 mRNA	Antisense oligonucleotide	Trabedersen	Pancreatic ductal adenocarcinoma and malignant pleural mesothelioma	NCT06079346 (phase 2/3) and NCT05425576 (phase 2)
TGF-β2 mRNA	Antisense oligonucleotide	TASO-001	Solid tumors	NCT04862767 (phase 1)
Furin	ShRNA	Vigil	Ovarian cancer	NCT02346747 (phase 2)
Latent TGF-β complex	Monoclonal antibody	SRK-181	Solid tumors	NCT04291079 (phase 1)
GARP	Monoclonal antibody	HLX60	Solid tumors and lymphoma	NCT05483530 (phase 1) and NCT05606380 (phase 1)
TGF-β2	Dietary supplement	Modulen	IBD	NCT04921033 (phase 3), NCT04777656 (phase 3), and RBR-955md27
TGF-β	Monoclonal antibody	NIS793	Pancreatic cancer, colorectal cancer, and MDS	NCT04935359 (phase 3), NCT04390763 (phase 2), NCT04952753 (phase 2), NCT05417386 (phase 1), and NCT04810611 (phase 1)
TGF-β	Monoclonal antibody	SAR439459	Multiple myeloma and osteogenesis imperfecta	NCT04643002 (phase 1/2) and NCT05231668 (phase 1)
TGF-β	Ligand trap	Bintrafusp alfa	Solid tumors	NCT05061823 (phase 3), NCT03436563 (phase 2), NCT04396886 (phase 2), NCT05005429 (phase 2), NCT04708470 (phase 1/2), NCT04574583 (phase 1/2), etc.
TβR	Dominant-negative TβR	TGF-β-resistant cytotoxic T lymphocytes	Lymphoma	NCT00368082 (phase 1)
TβRI	Kinase inhibitor	Galunisertib	Nasopharyngeal carcinoma, prostate cancer, colorectal cancer, and glioma	NCT04605562 (phase 2), NCT02452008 (phase 2), NCT02688712 (phase 2), NCT01582269 (phase 2), NCT05700656 (phase 1/2), and NCT01682187 (phase 1)
TβRI	Kinase inhibitor	Vactosertib	Solid tumors and myeloproliferative neoplasm	NCT04515979 (phase 2), NCT04064190 (phase 2), NCT05436990 (phase 2), NCT04103645 (phase 2), NCT05588648 (phase 1/2), NCT03802084 (phase 1/2), etc.
TβRI	Kinase inhibitor	LY3200882	Solid tumors	NCT02937272 (phase 1)

### Alteration of TGF-β biosynthesis

#### Targeting TGF-β mRNAs

Trabedersen (AP 12009 or OT-101) is an antisense oligonucleotide complementary to human TGF-β2 mRNA and can specifically inhibit TGF-β2 biosynthesis. It is hypothesized that trabedersen mainly acts by reversing TGF-β2-mediated immunosuppression to facilitate immune responses against tumors. A phase 2b clinical trial showed no advantage in early tumor control rate but in long-term survival rate for glioma patients treated with trabedersen in comparison with standard chemotherapy. Tumor responses which continued to increase long after discontinuation in the study suggested that the clinically relevant beneficial effects of trabedersen might increase over time. Moreover, compared with the standard chemotherapy group, drug-related or possibly drug-related adverse events in the trabedersen group were less common and mostly nervous system disorders. The study also indicated that the optimal dose of trabedersen is 10 µM, as both its efficacy and safety tended to be superior to the 80 µM dose, although the mechanism for this counterintuitive result has not been fully understood.^873^ TGF-β1 antisense oligonucleotides or small interfering RNAs (siRNAs) were also developed and evaluated in different pre-clinical models, suggested as potential therapeutic strategies for tuberculosis,^874,875^ wound scarring,^876,877^ and several renal diseases.^878–881^

TGF-β antisense gene-modified tumor cell vaccines are designed to exhibit increased immunogenicity due to reduced TGF-β expression in the tumor cells that comprise the vaccines. Vaccine Lucanix (belagenpumatucel-L) made from allogeneic non-small cell lung cancer (NSCLC) cell lines was well tolerated and brought survival advantages to NSCLC patients who were randomized within 12 weeks of completion of platinum-based chemotherapy and in those who had received prior radiation, as shown in a phase 3 trial which, however, failed to demonstrate a significant increase in survival in the overall patient population.^882^ TGF-β antisense-modified autologous tumor cell vaccines have also been tested in advanced glioma and other solid tumors, respectively, in two phase 1 studies in which enhanced anti-tumor activity and improved survival were observed.^34,883^ Notably, in the study among glioma patients, the most common treatment-related adverse events were delayed-type hypersensitivity-like reactions observed at the sites of the second and subsequent vaccinations in all patients. Some of these patients also experienced transient, flu-like symptoms consisting of musculoskeletal aches and pains and fatigue during the course of treatment.^34^

#### Targeting furin

Convertase furin is a therapeutic target participating in the post-translational processing of TGF-β. Vigil (FANG or Gemogenovatucel-T) is an autologous tumor cell vaccine incorporating a plasmid encoding granulocyte-macrophage colony-stimulating factor (GMCSF) and a bifunctional short-hairpin RNA (shRNA) targeting the expression of furin. A phase 1 study confirmed its safety and efficacy in various advanced solid tumors, with significant survival differences noted between patients who received less than four vaccinations and those who received no less than four vaccinations.^884^ A later phase 2b trial also demonstrated significant clinical benefit in homologous recombination proficient ovarian cancer (NCT02346747).^885^ Both studies reported no treatment-related serious adverse events, while the most common grade one and two adverse events related to study medication were local reactions at the injection site.

### Alteration of TGF-β activation

#### Targeting latent TGF-β complex

SRK-181 is an antibody that selectively binds to latent TGF-β1 to inhibit its activation. Co-administration of SRK-181 and anti-PD-1 antibody induced profound anti-tumor responses and survival benefit in mice, with increased infiltrating CD8+ T cells and decreased immunosuppressive myeloid cells observed in tumors refractory to anti-PD-1 treatment.^886^ The selective blockade of TGF-β1 by SRK-181 neither caused cardiac valvulopathy in rats as pan-TGFβ inhibitors might do nor did it induce cytokine release in human peripheral blood. Moreover, SRK-181 showed no effect on human platelet aggregation, activation, and binding.^886,887^ The favorable safety profile displayed in these preclinical assessments supports the ongoing phase 1 trial of SRK-181 in patients with advanced cancers (NCT04291079).

#### Targeting GARP

GARP expressed by Tregs, platelets, and endothelium functions to tether latent TGF-β complex to the cell surface for activation. Anti-GARP monoclonal antibody PIIO-1 proved to be an effective and safe strategy to block TGF-β activation in preclinical models, for it specifically bound to ligand-free GARP on Tregs but lacked recognition of GARP-latent TGF-β complex on platelets, actually avoiding the risk of platelet-related toxicities such as thrombocytopenia. More importantly, PIIO-1 showed therapeutic efficacy against both GARP+ and GARP- cancers alone or in combination with anti-PD-1 antibody, by preventing T cell exhaustion and enhancing CD8+ T cell migration into the TME in a C-X-C motif chemokine receptor 3 (CXCR3)-dependent manner.^888^

#### Targeting αV integrins

Integrins are regarded as the most important activators of TGF-β. Abituzumab (EMD 525797 or DI17E6) is an antibody against pan-αV integrins. In a phase 1/2 trial on KRAS wild-type metastatic colorectal cancer (NCT01008475), the progression-free survival (PFS) and response rates were similar among all groups in the intent-to-treat population comprising all patients randomized, although a trend toward improved overall survival (OS) was observed in the groups that received abituzumab treatment. However, exploratory analysis suggested that in patients with high αVβ6 expression, PFS and response rates might be increased with abituzumab therapy.^889^ This pan-αV integrin inhibitor was also found to inhibit prostate cancer-associated bone lesion formation in a randomized phase 2 trial (NCT01360840), although PFS was not significantly extended.^890^ Recently, abituzumab has been investigated in SSc-associated interstitial lung disease in a phase 2 trial (NCT02745145). However, the study was terminated prematurely due to slow enrollment and no meaningful conclusions could be drawn due to a small sample size.^891^ The most commonly reported treatment-related adverse events of abituzumab included fatigue, headache, gastrointestinal disorders, as well as abnormal biochemistry and hematology values.^889,890,892^

Cilengitide (EMD 121974, NSC 707544) is a selective αvβ3 and αvβ5 integrin inhibitor which has been evaluated for therapeutic efficacy in NSCLC (NCT00842712),^893,894^ head and neck SCC (NCT00705016),^895^ glioblastoma (NCT00689221, NCT00813943, and NCT01124240),^896–903^ melanoma,^904^ pancreatic cancer,^905^ and prostate cancer^906,907^ in a series of phase 2 studies and one phase 3 study. Although cilengitide failed to demonstrate significant clinical benefits in these studies on tumors, it might be a novel treatment for fibrotic diseases as relevant preclinical studies suggested.^908,909^ Notably, the adverse events possibly related to cilengitide treatment included fatigue, arthralgia, lymphopenia, and gastrointestinal disorders.^893,897,899,900,904,906,907^ Furthermore, an inhibitor of pan-integrins and TGF-β known as GLPG-0187 was proved to enhance T cell killing of colorectal cancer cells in vitro, possibly by suppressing TGF-β-mediated PD-L1 upregulation.^910,911^

#### Targeting TSP-1

TSP-1 can directly activate all three TGF-β isoforms independent of other activators or cellular activity. The conserved LSKL sequence in LAP which is recognized by TSP-1 can be synthesized as peptides to block TSP-1-mediated TGF-β activation. Pre-clinical studies suggested that treatment of LSKL or relevant tripeptide SRI31277 could be novel therapeutic strategies for various cardiovascular diseases,^912^ pulmonary diseases,^913^ renal diseases,^914–916^ nervous diseases,^917,918^ fibrotic diseases,^919–921^ wound healing,^922,923^ and tumors.^924–926^ Moreover, TSP-1 antisense oligonucleotides were successfully developed and applied to inhibit TGF-β activation in a rat model of mesangial proliferative glomerulonephritis, demonstrating a remarkable prevention against renal fibrosis.^927^

### Alteration of TGF-β signaling

#### Targeting TGF-β ligands

A TGF-β2-enriched polymeric dietary supplement known as Modulen (CT3211) was effective in inducing earlier remission of inflammatory bowel diseases (IBDs) including both Crohn’s disease and UC with significant improvements in endoscopic and histologic appearances, mucosal cytokine parameters, C-reactive protein (CRP) values, erythrocyte sedimentation rates (ESRs), serum albumin levels, as well as weight and height scores in the patients.^928–931^ Notably, an exclusive Modulen diet was more efficient than steroids to induce mucosal healing in children with Crohn’s disease, possibly due to its additional advantage in regulating intestinal microbiota (NCT00265772).^932,933^ Moreover, a pre-operative polymeric diet enriched with TGF-β2 was able to decrease post-operative complications after surgery for complicated ileocolonic Crohn’s disease.^934^ The side effects of Modulen were mild, including abdominal pain, flatulence, nausea, and vomiting.^928,932,934^ In mouse models, oral TGF-β supplementation also showed beneficial effects on food allergy prevention.^935–937^ In fact, it is believed that the presence of TGF-β in breast milk can protect the progeny from several allergic diseases such as asthma,^938^ eczema,^939^ and food allergy.^940^

Recombinant human TGF-β3 known as avotermin (Juvista) is a potential therapy for the improvement of cutaneous scarring. In a series of phase 1/2 studies (NCT00847925, NCT00847795, NCT00629811, NCT00432211, NCT00594581, and NCT00430326), visual assessment of scar formation revealed that, in contrast to placebo, intradermal avotermin could significantly improve total scar scores which were derived from a visual analog scale to assess how closely scars resembled normal skin. The results were further confirmed by histological assessments that scars treated with avotermin showed better organized ECM of the papillary and reticular dermis. The incidence of adverse events at wound sites, including infection, exudate, erythema, pain, burning, itching, and thickening was low and similar for avotermin and controls.^941–944^ Although the other two TGF-β isoforms, TGF-β1 and TGF-β2, showed no therapeutic activity of scarring, they were found to improve and accelerate the healing of cutaneous wounds in animal models as well as clinical patients.^304,306,307,317,321,334,945^ Moreover, TGF-β also showed therapeutic potential for tissue regeneration,^329,946,947^ inflammatory diseases,^676,687,948^ and influenza^949^ as shown in relevant preclinical models.

TGF-β neutralizing antibodies and ligand traps can block the binding of TGF-β to its receptors. Fresolimumab (GC1008), a monoclonal antibody that neutralizes all three TGF-β isoforms demonstrated acceptable safety and preliminary evidence of anti-tumor activity in a phase 1 study on advanced malignant melanoma and renal cell carcinoma (NCT00356460).^950^ In a phase 2 trial (NCT01401062), a higher dose of fresolimumab is associated with longer median OS as well as improved peripheral blood mononuclear cell counts and boosted central memory CD8+ T cell levels in metastatic breast cancer patients receiving radiotherapy.^951^ Fresolimumab also showed therapeutic effects on SSc with decreased biomarkers of skin fibrosis and improved clinical symptoms in the patients in a phase 1 study (NCT01284322).^952^ Moreover, a phase 1 study evaluated the safety of fresolimumab in patients with treatment-resistant primary focal segmental glomerulosclerosis and the good tolerability supported additional evaluation in larger randomized dose-ranging clinical trials.^953^ Notably, the major drug-related adverse events of fresolimumab were skin disorders, bleeding episodes, and anemia. Skin toxicity was particularly significant and tumor patients assigned to high doses of treatment even developed skin tumors, including keratoacanthoma, basal cell carcinoma, and SCC.^950–954^ Another anti-TGF-β monoclonal antibody known as NIS793 was well tolerated alone or in combination with anti-PD-1 antibody in patients with advanced solid tumors in a phase 1 study (NCT02947165). Treatment-related adverse events of all patients in the study were mostly skin toxicity and gastrointestinal events, and no dose-limiting toxicities were observed during dose escalation. Notably, biomarker analyses in the study showed evidence of systemic target engagement, local signaling inhibition, and tumor immune activation.^955^ Apart from tumors, a recombinant human anti-TGF-β1 antibody known as CAT-192 was evaluated in the treatment of early-stage diffuse cutaneous SSc but showed no evidence of efficacy in the pilot phase 1/2 study. The most commonly reported adverse events in the study affected the gastrointestinal, musculoskeletal, respiratory, and skin systems, but none of them were considered to be related to the treatment.^956^ Moreover, a phase 2 study assessing the safety and efficacy of TGF-β1 monoclonal antibody in patients with diabetic nephropathy was terminated early for futility (NCT01113801). The frequencies of the various categories of adverse effects in this study were generally similar between the treatment and placebo groups.^957^ Furthermore, monotherapy of a selective TGF-β1/3 trap known as AVID200 in a population of patients with an advanced stage of myelofibrosis in a phase 1b trial resulted in limited toxicity as well as improvements in spleen size, symptom benefit, and platelet counts (NCT03895112). Remarkably, platelet count increase was a therapeutic effect not observed with other myelofibrosis therapies, suggesting a potential advantage of AVID200 treatment. Adverse events that occurred during the study regardless of attribution mainly included pruritus, fatigue, abdominal pain, anemia, and thrombocytopenia.^958^ Additionally, other potential applications of neutralizing TGF-β antibodies suggested by pre-clinical studies include wound healing,^334,959,960^ prostatic hyperplasia,^961^ pulmonary diseases,^962,963^ cardiovascular diseases,^564,964^ musculoskeletal diseases,^965–968^ inflammatory diseases,^969,970^ and Chagas disease (*Trypanosoma cruzi* infection).^971^

Bifunctional antibody-ligand traps containing the extracellular domain of TβRII can target both TGF-β and immune checkpoints. In preclinical studies, both the anti-CTL associated protein (CTLA)-4-TβRII chimera and the anti-PD-L1-TβRII chimera exhibited superior anti-tumor efficacy compared with their parent immune checkpoint inhibitors.^972^ Bintrafusp alfa (M7824), a bifunctional fusion protein targeting both TGF-β and PD-L1 was assessed in several phase 1 trials (NCT02699515, NCT02517398, NCT02699515, and NCT04247282). The results showed that bintrafusp alfa had encouraging efficacy in NSCLC,^973^ gastric cancer,^974^ biliary tract cancer,^975^ as well as human papillomavirus (HPV)-unrelated head and neck cancer in which enhanced tumor antigen-specific immunity has been observed.^976^ Similar to fresolimumab, the treatment-related adverse events of bintrafusp alfa included fatigue, colitis, bleeding, anemia, hypokalemia, lipase increase, hepatic function abnormalities, as well as several skin disorders from rash, hyperkeratosis, to keratoacanthoma and SCC.^973–977^ BR102 is another bifunctional fusion protein simultaneously targeting PD-L1 and TGF-β. The efficacy and safety of BR102 demonstrated in preclinical characterization supported its further clinical development for anti-cancer therapy.^978^ Notably, the bifunctional antibody-ligand traps have inspired the development of chimeric antigen receptor (CAR)-T cells secreting bispecific trap protein, which co-targets PD-1 and TGF-β to enhance anti-tumor efficacy as shown in mouse models.^979^

Furthermore, LAP, TβRIII (β-glycan), and decorin can bind to TGF-β as natural inhibitors. They have shown treatment effects in preclinical models of wound healing,^980–983^ cardiovascular diseases,^984–989^ nervous diseases,^990–992^ renal diseases,^993–996^ fibrotic diseases,^997–1000^ tuberculosis,^1001^ and tumors^1002–1005^ and thus warrant further development.

#### Targeting TβRs

TGF-β-insensitive CAR-T cells armored with dominant-negative TβRII showed preliminary evidence for early anti-tumor function in prostate cancer, including a biomarker decline among approximately 30% of the patients in a phase 1 trial (NCT03089203). This strategy which is considered generally feasible, despite no partial response being observed in the study, and safe, with study-related serious adverse events mostly being cytokine release syndrome, warrants further validation and investigation.^1006^ Dominant-negative TβRII can also enhance the anti-tumor efficacy of DC vaccines, manifested by powerful tumor-specific CTL responses, inhibited tumor development, and prolonged survival times in mouse models.^1007,1008^ Moreover, dominant-negative TβRII showed great potential for reducing hypertrophic scars as in rabbit ear models.^1009^

Many small-molecule inhibitors have been developed to suppress the kinase activity of TβRI. In a series of phase 2 studies, a TβRI kinase inhibitor known as galunisertib (LY2157299) showed preliminary efficacy in patients with myelodysplastic syndromes (MDS) (NCT02008318),^1010^ NSCLC (NCT02423343),^1011^ hepatocellular carcinoma (NCT01246986),^1012,1013^ rectal cancer (NCT02688712),^1014^ and pancreatic cancer,^1015^ but failed to demonstrate clinical benefit in patients with glioma (NCT01582269 and NCT01220271).^1016,1017^ The most common adverse events related to galunisertib treatment included fatigue, pyrexia, anemia, nausea, vomiting, diarrhea, and abdominal pain.^1010,1013,1017^ Despite comprehensive cardiovascular monitoring for galunisertib did not detect medically relevant cardiac toxicity in cancer patients,^1018^ galunisertib-related uncontrolled cytokine release was reported in patients with advanced solid tumors in a phase 1 trial (NCT01646203).^1019^ Other TβRI kinase inhibitors such as SM16, SD-208, NP-40208, SB-431542, LY3200882, LY364947, and vactosertib (EW-7197) also showed therapeutic potential in pre-clinical studies on tumors^1020–1026^ as well as many other diseases such as cardiovascular diseases,^565,1027–1030^ renal diseases,^1031^ ophthalmic diseases,^1032^ skeletal diseases,^1033^ fibrotic diseases,^1034–1036^ inflammatory diseases,^1037–1039^ Chagas disease,^1040,1041^ coronavirus disease 2019 (COVID-19),^1042^ and wound healing.^1043–1045^

#### Targeting SMADs

An oral SMAD7 antisense oligonucleotide known as mongersen (GED-0301) showed promising results in patients with active Crohn’s disease in phase 1 and 2 phase trials, but further phase 3 study failed due to lack of clinical benefit (EudraCT 2009-012465-66, EudraCT 2011-002640-27, and NCT02596893).^1046–1048^ Meanwhile, SMAD3 antisense oligonucleotide treatment was found to improve flexor tendon repair in mice and might have possible therapeutic applications in clinical practice.^877^

Moreover, a small-molecule SMAD3 inhibitor known as specific inhibitor of SMAD3 (SIS3) has shown pre-clinical therapeutic efficacy in wound healing,^1049^ cardiovascular diseases,^569,1050,1051^ nervous diseases,^1052^ renal diseases,^1053,1054^ skeletal diseases,^1055^ fibrotic diseases,^1056,1057^ inflammatory diseases,^1039,1058^ type 2 diabetes,^1059,1060^ and tumors,^1061,1062^ suggesting a novel approach that could be further tested to treat clinical patients.

Furthermore, several SMAD-binding peptide aptamers have been developed to selectively inhibit the binding between SMADs and their interacting factors.^1063^ An aptamer containing the SMAD-binding domain of transcription factor lymphoid enhancer-binding factor 1 (LEF1) can suppress tumor cell proliferation by inhibiting the interaction between SMAD4 and LEF/T cell-specific factor (TCF) to suppress MYC expression.^1064^ Other aptamers that bind specifically to R-SMADs through the SMAD-binding domain from SARA can impair the formation of functional SMAD oligomers to inhibit TGF-β-induced EMT.^1065,1066^ Moreover, aptamers that disrupt the interaction between SMAD and transcription coactivator yes-associated protein (YAP) have been designed for bone tumor therapy.^1067^

## Conclusions and future perspectives

TGF-β signaling is so extensively and indispensably involved in a large number of biological processes that it has attracted great interest and attention over the past decades during which relevant knowledge has exploded in the fields of health, disease, and therapeutics. However, there are still some specific issues that have not been fully elucidated, while some previous knowledge is facing updates and challenges.

Studies on embryonic development and wound healing have revealed the isoform-specific roles of TGF-β which remain poorly aware in other fields of research, as studies on immune homeostasis, fibrotic diseases, and tumor development so far have focused on the most abundant TGF-β1 isoform in particular. Since all TGF-β isoforms are believed to signal through the same receptors and downstream pathways, the causes of the differences in biological effects between isotypes have not been fully understood. Moreover, since a natural TGF-β heterodimer containing one TGF-β1 monomer and one TGF-β2 monomer has long been discovered,^12,1068^ it would be very interesting to identify and characterize novel TGF-β heterodimers in the future. Furthermore, with the discovery and study of TGF-β superfamily which also includes polypeptides structurally similar to TGF-β such as nodal, myostatin, inhibins, activins, Müllerian-inhibiting substance (MIS), bone morphogenetic proteins (BMPs), and growth and differentiation factors (GDFs), researchers have realized that TGF-β can also signal through pathways ‘specific’ to other TGF-β superfamily members, for example, via receptors ALK1/2/3 and transcription factors SMAD1/5/8.^1069–1073^ The significance of the signaling crosstalk within the TGF-β superfamily also warrants future exploration. Notably, Reblozyl (luspatercept or ACE-536), a ligand trap that contains the extracellular domain of human activin receptor type IIB (ActRIIB) to inhibit GDF11-mediated SMAD2/3 signaling has been approved by the US Federal Drug Agency (FDA) for the treatment of anemia in adult patients with β-thalassemia or with MDS.

As for TGF-β-targeting therapy, the efficacy and safety of treatment are always issues of concern. The current lack of systematic studies on the dural roles of TGF-β in wound healing, infectious diseases, and tumor development may hinder the development of related therapeutics. Given the extensive impacts of TGF-β on a lot of biological processes, the development of TGF-β isoform-specific therapies and SMAD-binding peptide aptamers is expected to cause less adverse effects through more precise targeting. Moreover, the identification of the applicable population for each therapeutic approach is also important for better efficacy and less toxicity. Serum and tissue levels of TGF-β have shown potential as predictors or indicators of the development,^1074–1077^ complication,^1078–1080^ response,^1081–1084^ recurrence,^1085–1087^ and outcomes^1088–1090^ of various kinds of diseases, meanwhile, bioinformatic tools of TGF-β signaling-related gene expression signatures have also been developed for patient stratification.^863,1091^ But so far, TGF-β or related factors as clinical biomarkers still need further development and assessment.

To summarize, this review focuses on the multiple roles of TGF-β in health and disease while emphasizing the mechanisms of TGF-β production, activation, signaling, as well as corresponding therapeutic strategies. These understandings might be instructive for the basic and applied research of relevant topics in the future.

## Data Availability

Not applicable.
